# Establishment and molecular profiling of a PDX model of a metachronous brain tumor in a patient with constitutional mismatch repair deficiency with biallelic MSH6 variant

**DOI:** 10.1002/ame2.70069

**Published:** 2025-08-29

**Authors:** Daniel Antunes Moreno, Bruna Minniti Mançano, Mirella Baroni, Eric Allison Philot, Felipe Antonio de Oliveira Garcia, Murilo Bonatelli, Flávia Escremim de Paula, Iara Viana Vidigal Santana, Gustavo Ramos Teixeira, Mauricio Yamanari, Luciane Sussuchi da Silva, André Escremim de Paula, Augusto Perazzolo Antoniazzi, Adrian Willig, Xiaobin Xing, Zhenyu Xu, Lucas Lourenço, Carlos Almeida Junior, Silvia Aparecida Teixeira, Rui Manuel Reis

**Affiliations:** ^1^ Molecular Oncology Research Center Barretos Cancer Hospital Barretos Brazil; ^2^ Department of Pediatric Oncology Barretos Cancer Hospital Barretos Brazil; ^3^ Department of Molecular Diagnosis Barretos Cancer Hospital Barretos Brazil; ^4^ Department of Pathology Barretos Cancer Hospital Barretos Brazil; ^5^ Barretos School of Health Sciences Dr Paulo Prata Barretos Brazil; ^6^ Department of Radiotherapy Barretos Cancer Hospital Barretos Brazil; ^7^ Department of Oncogenetics Barretos Cancer Hospital Barretos Brazil; ^8^ SOPHIA Genetics Rolle Switzerland; ^9^ ICVS/3B's PT Government Associate Laboratory Braga Portugal; ^10^ Life and Health Sciences Research Institute (ICVS), School of Medicine University of Minho Braga Portugal

**Keywords:** Brazil, constitutional mismatch repair deficiency, exome, high‐grade glioma, medulloblastoma, MSH6

## Abstract

**Background:**

Constitutional mismatch repair deficiency (CMMRD) is a rare disorder resulting from biallelic germline pathogenic variants in mismatch repair genes. This study described the molecular profile of two metachronous brain tumors and a patient‐derived xenograft (PDX) from a Brazilian child with CMMRD.

**Methods:**

After PDX development, methylation array, whole exome sequencing, and NanoString techniques were applied to describe the genetic landscape of CMMRD.

**Results:**

A 6½‐year‐old girl was diagnosed with Sonic Hedgehog (SHH)‐activated medulloblastoma and somatic *TP53*‐mutant. After surgery and radiochemotherapy, she remained free of disease progression. At 10 years and 3 months, she developed a diffuse pediatric‐type high‐grade glioma (dpHGG). The child had a family history of cancer, and subsequent investigation revealed a biallelic germline variant on *MSH6* (c.3556+1G>A) with the absence of protein expression in both normal and tumor tissue. A PDX model of the dpHGG was developed. The methylation profile confirmed the diagnosis of both brain tumors and PDX, refining the classification of dpHGG, Rtk1 subtype, subclass A, with an actionable alteration on Platelet‐derived growth factor receptor A *(PDGFRA)*. Exome analysis showed high tumor mutational burden, with 3019, 540, and 1049 pathogenic variants in the medulloblastoma, dpHGG, and PDX, respectively. Only the medulloblastoma exhibited microsatellite instability. The *CD24*, *CD47*, and *CD276* immune checkpoints had elevated messenger RNA levels, yet no programmed death ligand 1 expression was observed in CMMRD‐derived tumors.

**Conclusion:**

We report an extensive molecular profile of a CMMRD patient, and the developed PDX model can be applied to explore new therapeutic approaches for CMMRD‐associated brain tumors.

## Graphical abstract

1. A 6½‐year‐old girl was diagnosed with a medulloblastoma, SHH activated, subtype 3 and *TP53* mutant (somatic). After surgery and chemotherapy, she was monitored with quarterly magnetic resonance imaging (MRI) scans and remained free of disease for almost 4 years.
2. At 10 years and 3 months she was diagnosed with another primary brain tumor, a diffuse pediatric‐type high‐grade glioma (dpHGG), H3 wild type and isocitrate dehydrogenase (IDH) wild type, Rtk1 subtype, subclass A. A biallelic germline variant on *MSH6* (c.3556+1G>A) was detected, resulting in the lack of MSH6 protein expression (immunohistochemistry [IHC]).
3. After surgery of the dpHGG, a PDX of the constitutional mismatch repair deficiency (CMMRD)–derived brain tumor was developed in our animal facility.

We conducted molecular characterization of mismatch repair deficiency (MMRD)–associated brain tumors, including medulloblastoma, dpHGG, and patient‐derived xenografts(PDX) of dpHGG. The results are summarized in the table below the graphical abstract.

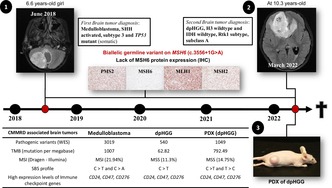

## 1 INTRODUCTION

Constitutional mismatch repair deficiency (CMMRD) is a rare genetic disease associated with biallelic inactivation in one of the four mismatch repair (MMR) genes: *PMS2* (60%), *MSH6* (20%–30%), *MLH1*, and *MSH2* (10%–20%).^1^, ^2^ CMMRD is associated with a very high incidence of a large spectrum of metachronous or synchronous cancers, including brain cancers, hematological neoplasms, and Lynch syndrome–related tumors.^2^, ^3^ CMMRD is reported to be the most aggressive and complex hereditary cancer syndrome and usually results in death early in life.^4^

Identifying patients with CMMRD has relevant implications in genetic counseling, and follow‐up is essential to improve patient outcomes.^5^ These tumors usually exhibit microsatellite instability (MSI) and high tumor mutational burden (TMB). Consequently, patients with CMMRD are expected to respond to immune checkpoint inhibitors, and this therapy showed improvement in patient outcomes and is currently recommended for the treatment of CMMRD‐associated tumors.^6^, ^7^ Due to the rarity of these cases, the genetic background of CMMRD remains relatively unexplored, particularly in the context of low‐ and middle‐income countries. Importantly, the lack of in vivo models hinders a comprehensive investigation of its biology. The predominant options consist of MMR knockout mouse lines, which, unlike patient‐derived xenograft (PDX) models, do not adequately preserve the high similarity in histopathological and molecular characteristics to the original tumor from patients with CMMRD.^8^

In this study, we present a Brazilian child who developed two metachronous brain tumors (medulloblastoma and high‐grade glioma [HGG]). Additionally, we provide an in‐depth exploration of the genetic landscape and immune checkpoint expression associated with both brain tumors. Moreover, we successfully developed a PDX model of pediatric‐type HGG.

## 2 METHODS

The detailed methodology is presented in Supplementary Material 1.

### 2.1 Case report

Briefly, the clinical data from the CMMRD patient and family were retrieved from the medical reports of Barretos Cancer Hospital (BCH), Brazil. The formalin‐fixed, paraffin‐embedded, and frozen tumor tissues and germ line DNA derived from blood samples were provided by the pathology department and the biobank^9^ of BCH, respectively. The molecular classification of both metachronous tumors (medulloblastoma and diffuse pediatric‐type high‐grade glioma [dpHGG]) was performed using the EPIC Bead Chip Infinium microarray version 2.0 kit (935 k) (Illumina, San Diego, CA, USA). Data were analyzed using the DKFZ/Heidelberg classifier version 12.8 (available at www.molecularneuropathology.org/). The medulloblastoma was also classified using a 21‐gene panel by nCounter (NanoString),^10^ and its genomic alterations were evaluated using the Trusight Tumor 15 NGS panel (Sophia DDM software).^11^

A PDX of the dpHGG was developed and validated at the BCH animal facility in NOD.Cg‐*Prkdc* ^scid^ *Il2rg* ^tm1Wjl^/SzJ (NSG) mice.^12^

### 2.2 PDX of dpHGG

A dpHGG, *H3* wild‐type, and *IDH1/2* wild‐type tumor sample obtained by surgical resection was used to develop the PDX model (OXP22). The fresh tumor tissue was collected during surgery at the Pediatric Oncology Department of BCH and was immediately processed for implantation. The sample was cut into small fragments (1–3 mm^3^) and subcutaneously engrafted into the flanks of 6‐ to 8‐week‐old NSG mice The Jackson Laboratory (JAX) using Matrigel (Corning), as previously described.^12^ The animals were housed in individually ventilated cages under specific pathogen‐free conditions, with controlled temperature and humidity, on a 12‐h light–dark cycle, and had free access to food and water (ad libitum).

The animals were monitored weekly for signs of morbidity and tumor growth. Body weight was recorded once a week, and tumor volume (*V*) was measured using a caliper and calculated using the formula *V* = *D* × *d* ^2^/2, where *D* is the longest diameter and *d* is the shortest diameter of the tumor. The time to initial tumor growth and successful first passage was ~200 days. Once the tumor reached ~300 mm^3^, the mice were euthanized. Tumors were aseptically collected and cut into small fragments (1–3 mm^3^) for subcutaneous engraftment into successive recipient mice, and also cryopreserved and fixed in formalin for histopathological and molecular analyses. All subsequent steps were performed using the aforementioned procedure.

All animal procedures were performed in accordance with institutional guidelines and approved by the Institutional Animal Care and Use Committee (45315021.0.0000.5437), ensuring compliance with ethical standards for the care and use of laboratory animals.

### 2.3 Genomic and immune profile

After DNA isolation from the frozen tumor tissues and blood of the patient, whole exome sequencing (WES) of blood, medulloblastoma, dpHGG, and PDX was performed at the SOPHiA Genetics Facility. The MSI status was assessed using distinct molecular approaches (multiplex polymerase chain reaction [PCR], Sophia Genetics MSI detection algorithm, and BaseSpace, Dragen, Illumina) and by evaluating MMR proteins (MLH1, PMS2, MSH6, and MSH2) using immunohistochemistry (IHC). Additionally, germline analysis was conducted with a custom hereditary panel of predisposing genes, including the mismatch repair *MSH1*, *MSH2*, *PMS2*, and *MSH6*.

The messenger RNA (mRNA) immune profile of the tumor tissue was assessed using the nCounter Human PanCancer Immune Profiling Panel (NanoString Technologies), which contains 730 immune oncology‐related genes as previously described.^13^ The use of patient's samples in the present study was approved by the ethics committee of BCH, and the parents previously signed the consent form (1775/2019, CAAE: 13585719.8.0000.5437, and 2139/2021, CAAE: 45315021.0.0000.5437).

The WES data are deposited in the European Genome‐phenome Archive (EGA): https://ega‐archive.org/datasets/EGAD50000000511.

## 3 RESULTS

### 3.1 Clinical presentation

A 6½‐year‐old girl with a cleft palate, exhibiting a persistent 1‐month history of headaches, vomiting, and convulsions, underwent a cranial computerized tomography at a local hospital in Maranhão state in Brazil. The imaging study revealed a distinctive lesion within the posterior fossa, specifically in the right cerebellar region, concurrent with hydrocephalus. Subsequently, the child was referred to the specialized oncology institution BCH, São Paulo, Brazil.

**FIGURE 1:**
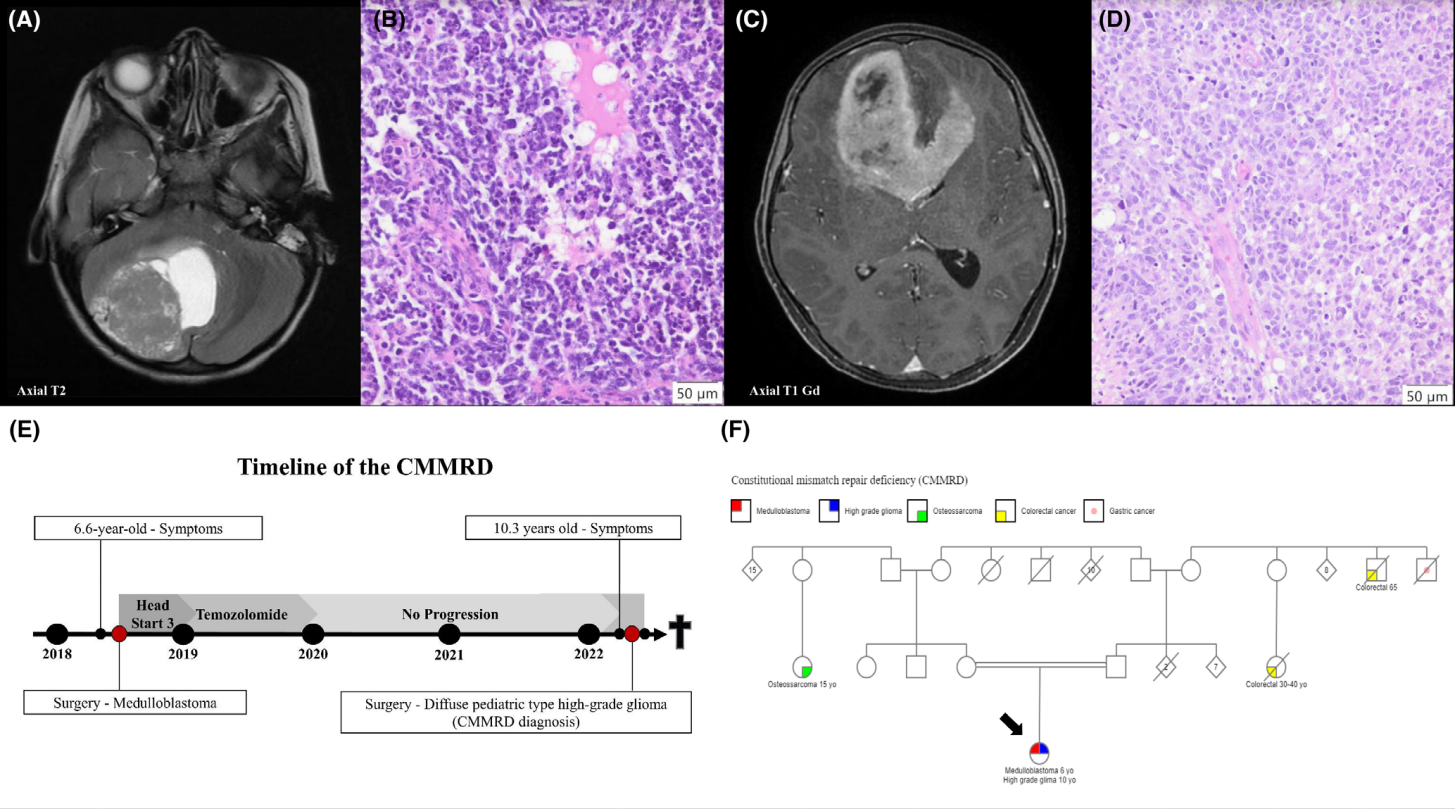
Brain magnetic resonance imaging (MRI) and histology of the medulloblastoma SHH‐activated and *TP53* mutant and the diffuse pediatric‐type high‐grade glioma (dpHGG), *H3* wild type, and *IDH* (isocitrate dehydrogenase) wild type (dpHGG). (A) Axial T2 MRI of medulloblastoma. (B) H&E (hematoxylin and eosin) stain of medulloblastoma. (C) Axial T1Gd MRI of the dpHGG. (D) H&E stain of the dpHGG. (E) Timeline illustrating the clinical course of the patient with CMMRD (constitutional mismatch repair deficiency). (F) Pedigree of the patient family with biallelic *MSH6* variant.

A comprehensive brain magnetic resonance imaging (MRI) examination was conducted at BCH to provide further insights into the patient's condition. The brain MRI revealed a significant solid and cystic expansive formation within the right cerebellar hemisphere, exhibiting midline displacement and exerting pressure on the fourth ventricle (Figure 1A). Histological examination, after subsequent surgery resection, revealed a small round blue cell neoplasm with abnormal pleomorphic nuclei and numerous apoptotic or mitotic figures (Figure 1B). IHC staining revealed positivity for synaptophysin, glial fibrillary acidic protein (GFAP) (focally), and p53 (focally), and retained INI1. Epithelial membrane antigen (EMA), cytokeratine8/18 (CK8/18), sal‐like protein4 (SALL4), and β‐catenin were negative (data not shown). These findings substantiated the diagnosis of medulloblastoma, histologically defined as anaplastic/large cell. The initial molecular classification showed an SHH‐activated medulloblastoma upon mRNA nCounter approach, with *TP53* mutation [c.993+1G>A/p.?] and [c.184G>T/p.(Glu62*)].

From June 2018 to December 2019, the child underwent treatment following the Head Start 3 chemotherapy protocol and temozolomide. Radiotherapy was administered until December 2018, involving a total dose of 36 Gy with an additional dose reaching 54 Gy. During medulloblastoma treatment, brain MRI exams were periodically performed, showing no evidence of cerebellar tumor progression from June 2018 to December 2021 (Figure S1A–E).

In March 2022, when the child was 10 years and 3 months old, ~3.7 years (44.5 months) after the initial diagnosis of medulloblastoma, a lesion was identified in the frontal lobe and corpus callosum. MRI showed a bifrontal expansive lesion, infiltrating the corpus callosum measuring 4.6 × 7.4 × 6.6 cm, showing a midline shift to the left and post‐contrast enhancement, restriction to diffusion increase in microvasculature and hemorrhagic component inside (Figure 1C; Figure S1F). After surgical removal of the lesion, the morphologic findings, such as glial cytology, necrosis, microvascular proliferation, and high mitotic index, suggested the diagnosis of infiltrative HGG (Figure 1D). When complete gross tumor resection of the HGG was performed, continuous bleeding was observed. Unfortunately, the child succumbed to the complications 3 days after the surgery. The clinical course of the CMMRD is summarized in Figure 1E.

IHC analysis revealed positivity for GFAP, oligodendrocyte transcription factor 2 (OLIG2), and p53 (strong and diffuse). IDH1^R132H^ was negative, and alpha‐thalassemia/mental retardation, X‐linked (ATRX) demonstrated the loss of nuclear expression. Ki‐67 index was high (80%). Sanger sequencing of *IDH1, IDH2*, and H3 histone, family 3A (*H3F3A)* was performed, and the tumor was classified as dpHGG, *H3* wild type, and isocitrate dehydrogenase (*IDH*) wild type.

### 3.2 Family cancer history and germline MSH6 variant detection

After the second tumor, a careful evaluation of the patient's family history was conducted at the BCH Oncogenetics Department. It showed the presence of several tumors, including two cases of colorectal cancer, one case of gastric cancer, and one case of osteosarcoma among family members. Additionally, the parents were cousins (Figure 1F).

**FIGURE 2:**
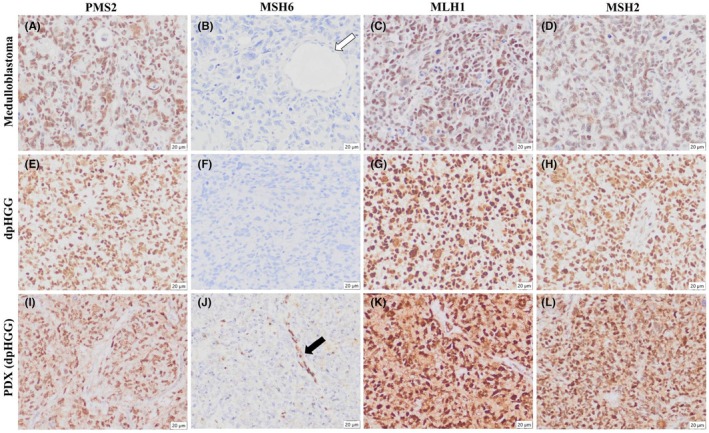
Immunohistochemistry (IHC) of the four DNA mismatch and repair‐associated proteins (PMS2, MSH6, MLH1, and MSH2) performed in the medulloblastoma: (A) PMS2, (B) MSH6, (C) MLH1, and (D) MSH2; in the dpHGG: (E) PMS2, (F) MSH6, (G) MLH1, and (H) MSH2; and in the PDX: (I) PMS2, (J) MSH6, (K) MLH1, and (L) MSH2. The white arrow indicates the endothelium cells with no MSH6 expression. The black arrow indicates the endothelium, which shows MSH6 expression in the xenograft.

Subsequent IHC analysis of the MMR‐associated proteins (PMS2, MSH6, MLH1, and MSH2) of both medulloblastoma (Figure 2A–D) and dpHGG (Figure 2E–H) was performed. It showed retained expression of all proteins, except MSH6, in both primary brain tumors (Figure 2B,F) and nontumor cells, such as endothelium (white arrow) (Figure 2B).

After these IHC results were obtained, the evaluation of germline variants on *MSH6* was performed in the proband blood using NGS revealing a homozygous *MSH6* variant (c.3556+1G>A) confirming its germline nature. Overall, this confirms the presence of a CMMRD. Parents and family members were invited for genetic testing to assess germline variants on *MSH6*, along with genetic counseling, but they did not attend.

The presence of the germline variant on *MSH6* was validated using different approaches, including NGS (Custom Hereditary Rare Cancer Solution kit) and WES.

### 3.3 dpHGG patient‐derived xenograft development

During the second surgery, and after parents signed informed consent and IRB approval was obtained, a part of the tumor was engrafted in an animal model for PDX development (Figure S2A). We successfully established the PDX model from the dpHGG, which reproduced the histological and IHC features of the primary tumor (Figure 2I–L; Figure S2B). Of note, mouse endothelium cells exhibited MSH6 expression (black arrow) (Figure 2J).

### 3.4 Methylation profile of CMMRD‐derived tumors

**FIGURE 3:**
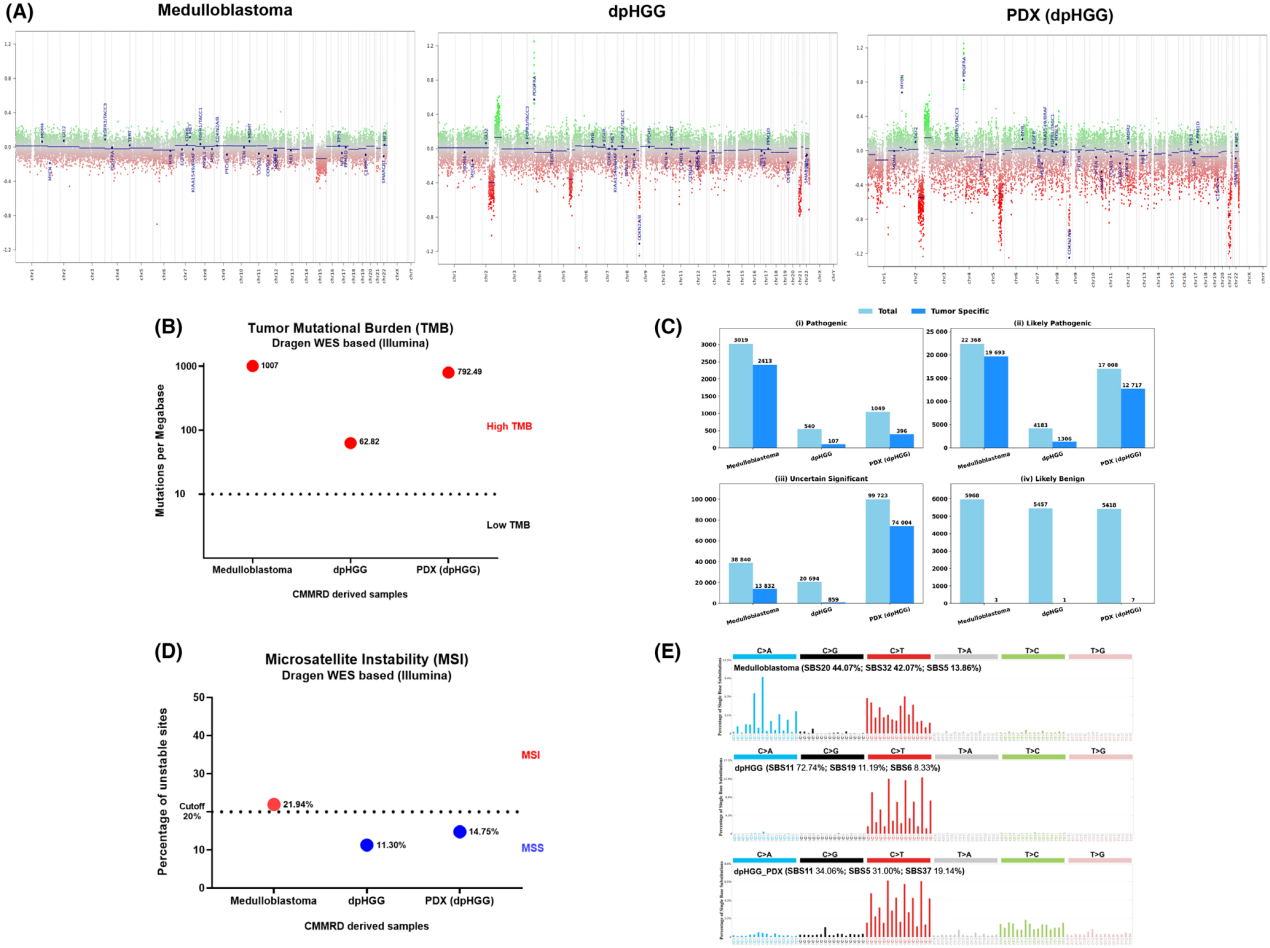
Genomic analysis of the CMMRD (constitutional mismatch repair deficiency)–derived brain samples: medulloblastoma, dpHGG, and PDX (dpHGG). (A) Copy number variation (CNV) prediction derived from methylation array. Gains and amplifications are represented by positive deviations (green), whereas losses are represented by negative deviations (red) from the baseline. (B) Tumor mutational burden (TMB) based on whole exome sequencing (WES), Illumina Dragen platform. (C) Total and tumor‐specific variants identified using WES of the CMMRD‐derived tumor samples. Bar plots represent the total number of alterations classified as (i) pathogenic, (ii) likely pathogenic, (iii) uncertain significance, and (iv) likely benign for both total and tumor‐specific variants. (D) Microsatellite instability (MSI) based on WES, Illumina Dragen platform. (E) Single base substitution (SBS) mutational profile. The main three signatures (SBS) are shown for each CMMRD tumor.

We performed the EPIC methylation array for the tumor classification and copy number alteration (CNA) analysis in the CMMRD‐derived brain tumors and in the PDX (Figure 3A). The diagnosis of both tumors was confirmed, with an additional refinement. Using the Heidelberg methylation brain classifier tool (version 12.8), the first brain tumor matched the medulloblastoma, SHH activated (score: 0.99), with 0.87 score, for subtype 3. The second primary brain tumor matched the class of dpHGG, *H3* wild type, and *IDH* wild type (score: 0.99), and Rtk1 subtype, subclass A (score: 0.99). The PDX of the dpHGG showed exactly the same classification observed in the primary tumor, with a score of 0.99 for dpHGG, Rtk1 subtype, subclass A.

The CNA showed that the medulloblastoma did not exhibit expressive alterations, except for chromosome 15 loss (Figure 3A). The primary dpHGG and the PDX of the dpHGG exhibited multiple CNAs, including losses and gain in chromosome 2, and losses in chromosomes 5 and 21. Moreover, both primary dpHGG and PDX of the dpHGG exhibited focal loss of *CDKN2A/2B* (Chr9) and *PDGFRA* amplification (Chr4) (Figure 3A).

### 3.5 Genomic profile of CMMRD‐derived tumors

The quality coverage of whole genome sequencing (WES) for samples is summarized in Table S1. We observed a high TMB in all samples, with 1007, 62.82, and 792.49 mutations per megabase (Mb) in the medulloblastoma, primary dpHGG, and PDX (dpHGG), respectively (Figure 3B). The median TMB described for pediatric malignancies is 1.7 mutations/Mb,^14^ and mismatch repair proficient pediatric tumors usually exhibit <10 mutations/Mb.^15^ Our somatic mutation analysis showed the presence of 2413 tumor‐specific pathogenic variants in the medulloblastoma, 107 in the primary dpHGG, and 396 in the PDX (dpHGG) (Table S2; Figure 3C). The analysis of the tumor DNA using WES showed a large number of variants in the CMMRD‐derived medulloblastoma, primary dpHGG, and PDX of the dpHGG, namely 3019, 540, and 1049 total pathogenic variants, respectively (Table S2; Figure 3C).

To describe the main pathogenic variants associated with central nervous system (CNS) tumors, we first selected 152 genes associated with CNS tumors and applied a virtual panel (Table S3). In the medulloblastoma, we observed 45 tumor‐specific pathogenic variants in 35 CNS tumor‐related genes, including *PTCH1, PTCH2*, and *SMO*, which are frequently altered in medulloblastomas (Table 1). In the primary dpHGG, we observed three tumor‐specific pathogenic variants, including the *ATRX, FOXO3*, and *TP53* (Table 1). In the PXD of the dpHGG, we observed pathogenic variants in 16 genes associated with CNS tumors (Table 1).

**TABLE 1:** Pathogenic variants identified in the medulloblastoma, dpHGG, and PXD (dpHGG) from the CMMRD patient.

CNS‐associated pathogenic variants found in CMMRD					
Gene	Coding consequence	Depth	Variant %	c.DNA	Protein
*Medulloblastoma*					
*ABL1*	Splice_acceptor_‐1	424	39.7	c.1424‐1G>T	p.(?)
*ALK*	Nonsense	488	5	c.4033G>T	p.(Gly1345*)
*APC*	Nonsense	519	13.6	c.4926T>A	p.(Tyr1642*)
*ARID1B*	Splice_acceptor_‐1	565	37.2	c.2841‐1G>T	p.(?)
*ARID1B*	Nonsense	688	19.1	c.6172G>T	p.(Gly2058*)
*ATR*	Splice_donor_+1	511	8.2	c.7349+1G>A	p.(?)
*ATR*	Nonsense	620	19.5	c.583C>T	p.(Gln195*)
*BCOR*	Nonsense	671	43.8	c.3763G>T	p.(Glu1255*)
*BRPF1*	Nonsense	686	45.5	c.2905G>T	p.(Glu969*)
*CDH1*	Splice_acceptor_‐1	199	47.3	c.1937‐1G>A	p.(?)
*CREBBP*	Nonsense	254	44.7	c.4380T>A	p.(Tyr1460*)
*DDX3X*	Splice_donor_+1	229	47.1	c.103+1G>A	p.(?)
*DDX3X*	Missense	257	49	c.1582C>T	p.(Arg528Cys)
*EZH2*	Nonsense	343	39.9	c.712G>T	p.(Glu238*)
*FBXW7*	Nonsense	297	42.9	c.478C>T	p.(Arg160*)
*FGFR1*	Nonsense	657	7	c.850C>T	p.(Gln284*)
*FUBP1*	Nonsense	235	5.8	c.679C>T	p.(Gln227*)
*GABRA6*	Nonsense	263	14.4	c.582C>A	p.(Tyr194*)
*JAK3*	Splice_acceptor_‐1	802	5	c.2351‐1G>A	p.(?)
*KDM6A*	Nonsense	306	8.1	c.3163C>T	p.(Gln1055*)
*KDR*	Nonsense	583	5.7	c.3927T>G	p.(Tyr1309*)
*KRAS*	Missense	284	6.6	c.40G>A	p.(Val14Ile)
*MGMT*	Nonsense	675	53.7	c.313G>T	p.(Glu105*)
*MGMT*	Nonsense	537	48.3	c.25G>T	p.(Glu9*)
*MSH2*	Nonsense	462	38.2	c.2131C>T	p.(Arg711*)
*MYB*	Splice_acceptor_‐1	241	5.9	c.307‐1G>T	p.(?)
*NF1*	Nonsense	405	15.6	c.910C>T	p.(Arg304*)
*NOTCH2*	Splice_acceptor_‐1	244	5.6	c.5003‐1G>A	p.(?)
*NOTCH2*	Nonsense	376	7.6	c.2299C>T	p.(Gln767*)
*PIK3C2G*	Nonsense	119	16.7	c.3918G>A	p.(Trp1306*)
*PTCH1*	Nonsense	353	24.8	c.678T>A	p.(Cys226*)
*PTCH2*	Splice_donor_+1	661	14.5	c.935+1G>A	p.(?)
*PTPN11*	Nonsense	508	5.8	c.1522G>T	p.(Glu508*)
*SETD2*	Splice_acceptor_‐1	269	21.6	c.7432‐1G>T	p.(?)
*SETD2*	Nonsense	293	46.2	c.5290C>T	p.(Gln1764*)
*SMARCA2*	Missense	189	25	c.2486C>T	p.(Thr829Ile)
*SMO*	Nonsense	379	7	c.768G>A	p.(Trp256*)
*TBR1*	Nonsense	633	5.4	c.122C>A	p.(Ser41*)
*TCF4*	Missense	798	45.9	c.1727G>A	p.(Arg576Gln)
*TP53*	Splice_donor_+1	373	44.1	c.993+1G>A	p.(?)
*TP53*	Nonsense	824	43.3	c.184G>T	p.(Glu62*)
*TSC2*	Splice_donor_+1	911	19.6	c.2220+1G>A	p.(?)
*TSC2*	Nonsense	938	37	c.3281C>A	p.(Ser1094*)
*TSC2*	Nonsense	690	42.8	c.1221C>G	p.(Tyr407*)
*TSC2*	Splice_acceptor_‐1	377	24.8	c.482‐1G>T	p.(?)
*dpHGG*					
*ATRX*	Splice_donor_+1	721	39.5	c.3943+1G>A	p.(?)
*FOXO3*	Nonsense	555	38.4	c.1354C>T	p.(Gln452*)
*TP53*	Splice_acceptor_‐1	876	40.4	c.994‐1G>A	p.(?)
*PDX (dpHGG)*					
*ARID1B*	Frameshift	530	11.8	c.621_625del	p.(Leu208Hisfs*22)
*ARID1B*	Inframe_9	960	9.2	c.1043_1051dup	p.(Ala348_Ala350dup)
*ARID1B*	Inframe_9	904	13.2	c.1003_1004insCGGCAGCAG	p.(Ala334_Gly335insAlaAlaAla)
*ARID2*	Nonsense	679	10.1	c.5503C>T	p.(Gln1835*)
*ATR*	Splice_donor_+2	977	5.6	c.7349+2T>C	p.(?)
*ATRX*	Splice_donor_+1	712	48.5	c.3943+1G>A	p.(?)
*DICER1*	No‐start	812	10.2	c.1A>T	p.(Met1?)
*E2F2*	Frameshift	921	8.9	c.231_232del	p.(Leu78Alafs*23)
*FOXO3*	Nonsense	550	50.6	c.1354C>T	p.(Gln452*)
*KDM6A*	Inframe_6	793	7.1	c.33_38dup	p.(Ala16_Ala17dup)
*PTCH1*	Nonsense	1211	5.7	c.4017G>A	p.(Trp1339*)
*TP53*	Splice_acceptor_‐1	990	51.3	c.994‐1G>A	p.(?)

Abbreviations: CMMRD, constitutional mismatch repair deficiency; CNS, central nervous system; dpHGG, diffuse pediatric‐type high‐grade glioma.

Of note, all variants of primary dpHGG were also observed in the PDX of the dpHGG (Table 1). We also found additional variants in the dpHGG‐PDX model, not observed in the dpHGG lesion, such as *ARID1B, ARID2, ATR, DICER1, E2F2, KDM6A*, *PTCH1*, and *TP53* (Table 1).

### 3.6 MSI analysis

The detection of MSI in the context of CMMRD, mainly due to *MSH6* mutations, is reportedly challenging.^15^, ^16^ Using a PCR method based on the six quasi‐monomorphic mononucleotide repeat markers, no MSI was observed in the medulloblastoma, the primary dpHGG, and the PDX (dpHGG) (Table S4). Using the Sophia Genetics algorithm in the WES for MSI screening, we observed microsatellite stability (MSS) in the primary dpHGG (median score 0.003) and MSI‐low confidence in the PDX (dpHGG) (median score 0.005). We could not perform this analysis in the medulloblastoma (Table S4). Finally, using the Illumina's Dragen App pipeline for WES, only the medulloblastoma was considered MSI‐high based on the cutoff of 20% recommended for this analysis, whereas the primary dpHGG and the PDX (dpHGG) showed MSS profiles (Figure 3D; Table S4).

### 3.7 Mutational signatures

Figure 3E shows that the main substitutions that characterized the single base substitution (SBS) profile are C > T and C > A for medulloblastoma, C > T for primary dpHGG, and C > T and T > C for the PDX (dpHGG). In the medulloblastoma, we observed SBS20 (44.07%), SBS32 (42.07%), and SBS5 (13.86%). The primary dpHGG exhibited SBS11 (72.74%), SBS19 (11.19%), SBS6 (8.33%), SBS5 (5.93%), and SBS1 (1.81%). The PDX of the dpHGG presented SBS11 (34.06%), SBS5 (31.0%), SBS37 (19.14%), SBS19 (7.37%), and SBS54 (4.95%) (Figure S3). The indel (ID) spectra observed for the CMMRD‐associated brain tumors mainly consisted of single T deletion in a repeat with ≥6 bp and single T insertion in a repeat with ≥5 bp both in T‐mononucleotide repeats (Figure S4). The contributions of each assigned ID signature are presented in Table S5. Figure S5 shows a heat map illustrating the contribution of each SBS and ID signature across individual tumor samples (Figure S5A); the bar plots present the total number of SBS mutations per sample (Figure S5B), and the bar plots show the total number of ID (indel) mutations per sample (Figure S5C).

### 3.8 Immune checkpoint expression profile in tumors in CMMRD

**FIGURE 4:**
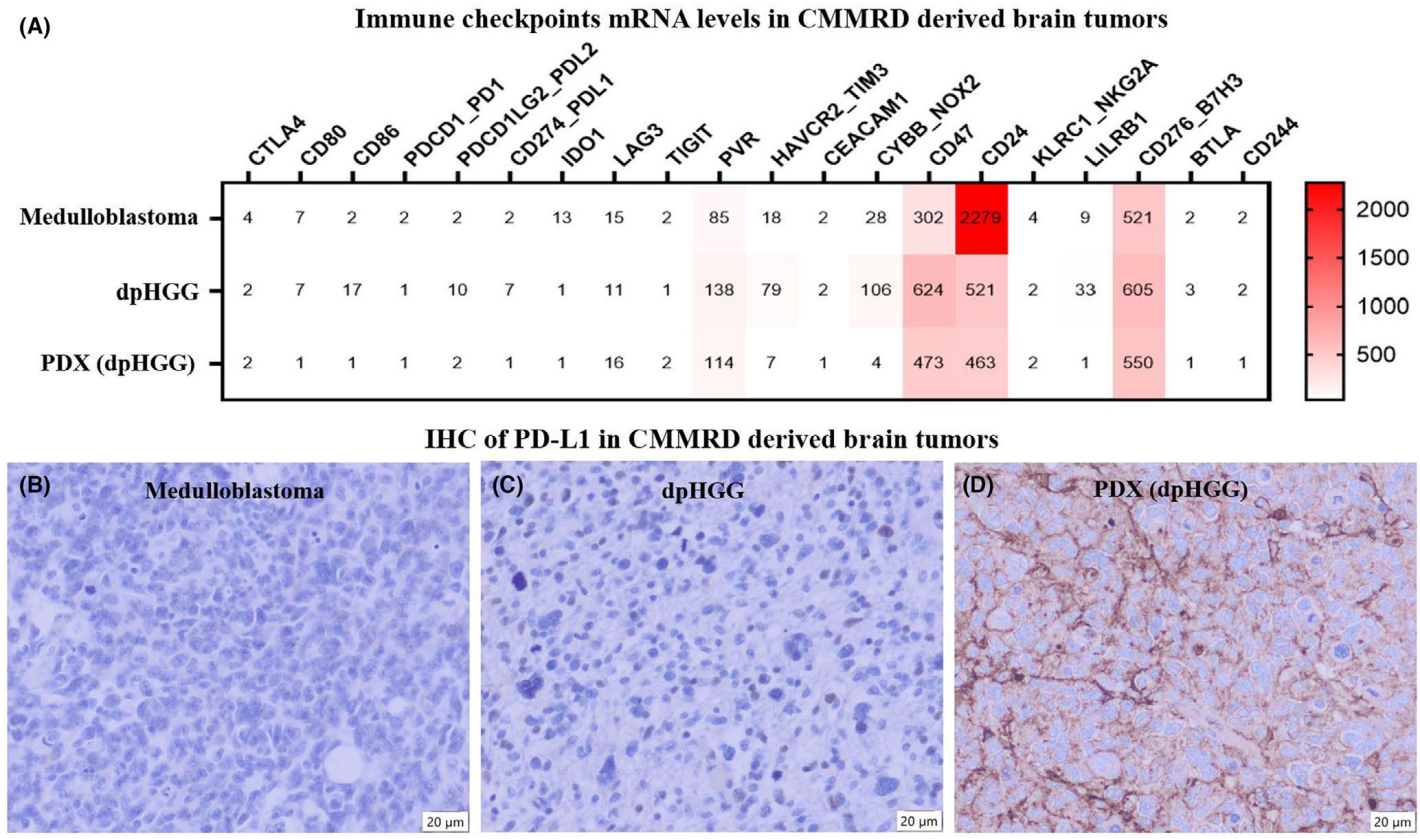
Immune checkpoint expression profile in CMMRD (constitutional mismatch repair deficiency)–derived tumor samples. (A) Immune checkpoint mRNA (messenger RNA) levels in the medulloblastoma (MB), dpHGG, and PDX of the dpHGG. The dashed line shows the mRNA levels of CD774 (PD‐L1 [programmed death ligand 1]) in the CMMRD‐derived tumor samples. (B) Immunohistochemistry of PD‐L1 performed in the medulloblastoma, (C) dpHGG, and (D) PDX of the dpHGG.

We evaluated the mRNA levels of 20 inhibitory immune checkpoint genes and receptors using nCounter technology: *CTLA4, CD80, CD86, PDCD1* (PD1), *PDCD1LG2* (PDL2), *CD274* (PD‐L1), *IDO1, LAG3, TIGIT, PVR, HAVCR2* (TIM3), *CEACAM1, CYBB* (NOX2), *CD47, CD24, KLRC1* (NKG2A), *LILRB1, CD276* (B7H3), *BTLA*, and *CD244* (Figure 4A). The canonical immune checkpoints associated with genes *CD274* (PD‐L1 [programmed death ligand 1]), *CTLA4*, and *PDCD1* (PD1) showed low mRNA levels in all samples (Figure 4A). High expression levels were observed only for the *CD24, CD47*, and *CD276* (B7H3) genes (Figure 4A).

IHC showed no PD‐L1 protein expression in the medulloblastoma (Figure 4B), in the primary dpHGG (Figure 4C), and in the PDX of dpHGG (Figure 4D), corroborating the low *CD274* mRNA levels. Of note, PD‐L1 expression in the PDX exhibited only positivity in mouse stroma cells (Figure 4D).

## 4 DISCUSSION

CMMRD is a rare and aggressive cancer predisposition syndrome caused by biallelic dysfunction of DNA mismatch and repair proteins. Children with CMMRD are typically diagnosed with cancer at 8–9 years old.^1^, ^2^ In the present study, we reported, to the best of our knowledge, the first Brazilian case of CMMRD associated with a biallelic loss of *MSH6* in a child who developed two metachronous brain tumors, an anaplastic *TP53‐*mutant SHH medulloblastoma at 6 years of age and a dpHGG, *H3* wild type, and *IDH* wild type (dpHGG) ~4 years later.

A recent study revealed that CNS tumors, comprising 51% of cases, is the predominant cancer type associated with CMMRD.^1^ We further developed the first reported PDX model of a dpHGG in a CMMRD context and performed an extensive molecular profile of both tumors and the PDX model.

Diagnosing CMMRD presents challenges, clinical manifestations usually overlap other human syndromes,^1^ the MSI patterns differ significantly from Lynch syndrome tumors,^15^ and detecting MSI in the context of CMMRD, particularly with *MSH6* mutations, proves to be intricate.^15^, ^16^ In fact, our initial analysis of MSI in both metachronous brain tumors, using a standard quasi‐monomorphic marker panel for colorectal cancer,^17^ showed an MSS phenotype. Moreover, the analysis of a larger panel based on Sophia Genetics MSI detection on the WES failed to detect the MSI status, and the Illumina's Dragen somatic pipeline evidenced MSI only in medulloblastoma.

It was the absence of MSH6 IHC, coupled with the detection of an *MSH6* germline variant (c3556+1G>A), high TMB, and family history, that confirmed CMMRD. A recent study from the CMMRD consortium shows the authors analyzed a series of 376 mismatch repair deficiency (MMRD)–associated childhood cancers and demonstrated that the low pass genomic instability characterization assay can detect MSI in 100% of MMRD cases.^15^ The sensibility of only 14% was demonstrated using the PCR five markers–based method, 80% using TMB, and 86% using IHC in MMRD childhood cancers.^15^ Moreover, high discordant results (36%) using IHC and MSI were observed, especially for *MSH6* variant carriers in Lynch syndrome–associated tumors.^16^ Recently another NGS‐based method called highly sensitive assessment of MSI showed high efficiency in detecting MSI in CMMRD‐derived samples.^18^ When it is not possible to perform these sensitive NGS‐based tests for detecting MSI, IHC testing and family history assessment are essential for detecting cases of MMRD. The *MSH6* germline variant (c3556+1G>A) identified in our proband is in a splice donor site, and this is the first description of this variant in CMMRD. The presence of parental consanguinity in our case is in line with previous studies that showed its presence in approximately half of CMMRD cases.^1^ Moreover, our family pedigree exhibited a high cancer prevalence, as it is reported (88%) among extended family members of CMMRD patients.^1^

Importantly, our PDX model of CMMRD showed exactly the same methylation classification of the primary tumor: dpHGG, Rtk1 subtype, subclass A. This specific subtype is enriched in Lynch syndrome or CMMRD‐associated HGG, and approximately 33% of cases harbor *PDGFRA* amplification, like our case.

To further gain insight into the biology of brain tumors in the context of CMMRD, we performed a WES analysis of primary tumors and PDX. Whereas the median TMB described for adult and pediatric malignances is 3.6 and 1.7 mutations/Mb, respectively,^14^ in the current investigation, we observed a considerable higher number of mutations per megabase in CMMRD‐associated tumors. The higher TMB (1007 mutations/Mb) was found in the medulloblastoma, followed by the PDX of the dpHGG (792.49 mutations/Mb) and the primary dpHGG (62.82 mutations/Mb). As expected for CMMRD‐associated tumors, we found a high number of pathogenic variants in the medulloblastoma (*n* = 3019), primary dpHGG (*n* = 540), and PDX from the dpHGG (*n* = 1049). This high number of pathogenic variants reflects the high TMB observed in CMMRD‐associated tumors.

To explore the CNS‐associated mutations in the CMMRD‐derived brain tumors, we evaluated a virtual panel of 152 genes implicated in the CNS tumors. As expected, recurrent mutations in SHH medulloblastomas, such as *PTCH1* and *SMO*,^19^ were also observed in our medulloblastoma. We also found a mutation in ATRX, frequently observed in the dpHGG.^20^

Interestingly, the PDX of dpHGG exhibited the same CNS‐associated variants observed in the primary dpHGG. The PDX exhibited some additional mutations, such as on *DICER1*, which could contribute to the increased mutational burden observed in the PDX compared with the primary dpHGG. These additional mutations observed in the PDX of the dpHGG, compared to the primary tumor, likely reflect biological evolution and subclonal selection of MMRD cells during the passages involved in establishing the animal model. It is also important to note that certain variants, such as *ATRX*, *DICER1*, and *PDGFR2* amplification, could serve as promising direct or indirect therapeutic targets. ATRX‐deficient cells, in particular, are thought to be more susceptible to PARP‐1 inhibition.^21^ DICER1 deficiency may activate the mitogen‐activated protein kinase (MAPK) signaling pathway, potentially increasing susceptibility to MAPK inhibitors.^22^ *PDGFRA* amplification has been shown to be a promising therapeutic target in pediatric gliomas.^23^, ^24^

Mutational signature analysis showed the MMRD‐related SBS20 in the medulloblastoma and SBS6 in the primary dpHGG. SBS1 occurred in both primary and PDX of the dpHGG and SBS5 in the three CMMRD‐associated tumors (clock‐like signatures); both signatures were reported in these pediatric cancer types.^25^ SBS11 related to temozolomide treatment was identified in the dpHGG, consistent with the treatment previously received during the treatment of the first brain tumor (medulloblastoma). SBS1, SBS5, SBS6, SBS20, SBS32, and SBS54 were also reported in CMMRD‐related HGG.^26^ The ID1 and ID2 signatures were identified in our samples. These are related to slippage during DNA replication, tend to occur in cancer samples with MMRD and MSI, and were found in the medulloblastoma and HGG tumors in pediatric cases.^25^

The presence of MMRD in solid tumors is believed to stimulate the production of neoantigens, which are traditionally recognized as predictive biomarkers indicating a favorable response to therapy with immune checkpoint inhibitors.^27^ Several studies reported the effectiveness of PD‐1 inhibitors such as nivolumab,^6^, ^28^ pembrolizumab,^3^, ^29^ and CTLA4 inhibitors (ipilimumab)^3^ in treating pediatric brain tumors associated with CMMRD. Nevertheless, a recent study reported that not all tumors associated with MMRD respond to anti‐PD‐1, anti‐PD‐L1, and anti‐CTLA4‐based immunotherapy.^30^ It is also important to note that some mismatch repair–deficient tumors should exhibit resistance to immune checkpoint blockade‐based immunotherapy.^31^ From a clinical standpoint, it is important to emphasize that in such cases, different immunotherapeutic approaches may lead to more effective outcomes for patients.

To interrogate the immune checkpoint profile of our CMMRD‐derived tumors, we evaluated the mRNA expression of 20 major inhibitory immune checkpoints. We found that canonical immune checkpoints, such as *CTLA4, PDCD*1 (PD1), and *CD274* (PD‐L1), are absent or exhibit a very low mRNA expression in the medulloblastoma, dpHGG, and PDX. The IHC expression of PD‐L1 was not detected, corroborating the mRNA expression levels. At variance, we observed high *CD24, CD47*, and *CD276* (B7H3) mRNA levels in all CMMRD‐derived tumors. A similar profile was recently reported on a series of 88 medulloblastomas.^32^ Moreover, we recently reported that high *CD276* mRNA levels are associated with the B7‐H3 protein levels, unfavorable outcomes, and metastasis in patients with medulloblastomas.^33^

Immunotherapies using anti‐CD24,^34^ anti‐CD47,^35^ and anti‐B7‐H3^36^ have shown promising antineoplastic effects. Preclinical studies showed efficacy of anti‐B7‐H3 immunotherapy for brain tumors.^37^ Clinical trials are investigating novel immunotherapies using CAR‐T cell and antibody–drug conjugates targeting B7‐H3 for refractory/recurrent brain cancers, including glioblastoma, diffuse intrinsic pontine glioma, medulloblastoma, ependymoma, and brain metastases.^37^

Future studies are needed to explore whether CD24, CD47, or CD276 (B7H3) inhibitors would constitute alternative immunotherapy approaches in CMMRD‐derived brain tumors. Moreover, dpHGG occurring within the context of CMMRD, frequently characterized by *PDGFRA* amplification, and PDGFRA inhibitors should be considered.

Of note, our PDX model of dpHGG in the context of a CMMRD exhibited, along with histopathological features, a similar molecular and immune checkpoint profile and, therefore, could constitute a valuable model for an in‐depth understanding of CMMRD biology and treatment options. An important limitation of our preclinical PDX model is that the mouse used is immunosuppressed, which facilitates engraftment but, on the contrary, limits experiments evaluating the efficacy of immunotherapies. Therefore, other preclinical models, such as PDX or mice with humanized immune systems, may be more suitable for studying the efficacy of specific immunotherapeutic approaches.

## 5 CONCLUSION

This is the first genetic and immune checkpoint landscape of a PDX model of the pediatric HGG in the context of a constitutive mismatch repair deficiency patient associated with germline biallelic *MSH6* inactivation, which could be an instrumental tool for exploring alternative therapeutic strategies. This preclinical model should contribute to improvements in personalized therapy for CMMRD‐associated gliomas.

## AUTHOR CONTRIBUTIONS

**Daniel Antunes Moreno:** Data curation; formal analysis; investigation; methodology; project administration; validation; visualization; writing – original draft; writing – review and editing. **Bruna Minniti Mançano:** Data curation; formal analysis; investigation; writing – original draft; writing – review and editing. **Mirella Baroni:** Data curation; formal analysis; investigation; methodology; writing – review and editing. **Eric Allison Philot:** Investigation; methodology; writing – review and editing. **Felipe Antonio de Oliveira Garcia:** Investigation; methodology; writing – review and editing. **Murilo Bonatelli:** Data curation; methodology; writing – review and editing. **Flávia Escremim de Paula:** Data curation; methodology; writing – review and editing. **Iara Viana Vidigal Santana:** Data curation; investigation; methodology; writing – review and editing. **Gustavo Ramos Teixeira:** Data curation; investigation; methodology; writing – review and editing. **Mauricio Yamanari:** Data curation; methodology. **Luciane Sussuchi da Silva:** Data curation; investigation; methodology; writing – review and editing. **André Escremim de Paula:** Data curation; methodology; writing – review and editing. **Augusto Perazzolo Antoniazzi:** Data curation; formal analysis; investigation; methodology; writing – review and editing. **Adrian Willig:** Funding acquisition; methodology; writing – review and editing. **Xiaobin Xing:** Funding acquisition; methodology; writing – review and editing. **Zhenyu Xu:** Funding acquisition; methodology; writing – review and editing. **Lucas Lourenço:** Data curation; investigation; writing – review and editing. **Carlos Almeida Junior:** Data curation; investigation; writing – review and editing. **Silvia Aparecida Teixeira:** Investigation; supervision; writing – review and editing. **Rui Manuel Reis:** Funding acquisition; investigation; project administration; supervision; writing – review and editing.

## FUNDING INFORMATION

The Brazilian Ministry of Health (MoH), National Council for Scientific and Technological Development‐ CNPq; and the Brazilian National Program of Genomics and Precision Health— Genomas Brasil (MS‐ SECTICS‐ Decit/CNPq 16/2023); Barretos Cancer Hospital (13/2021); Public Ministry of Labor Campinas (Research, Prevention, and Education of Occupational Cancer); São Paulo Research Foundation (FAPESP‐ 2021/07957–5)(Mirella Baroni), National Oncology Care Support Program (PRONON) and CNPq (444217/2023–1) (Daniel Antunes Moreno); CNPq Productivity (Rui Manuel Reis).

## CONFLICT OF INTEREST STATEMENT

The authors declare that they have no conflicts of interest.

## ETHICS APPROVAL AND CONSENT TO PARTICIPATE

Molecular analysis (1775/2019, CAAE: 13585719.8.0000.5437) and the PDX model development (2139/2021, CAAE: 45315021.0.0000.5437) were previously approved by the ethics committee of Barretos Cancer Hospital, and consent form was signed by parents.

## Supporting information


**Data S1.** Detailed description of the methods.

**Figure S1.** Brain magnetic resonance imaging (MRI) monitoring the clinical evaluation of the CMMRD (constitutional mismatch repair deficiency) patient. (A) Diagnosis of medulloblastoma in the left cerebellar hemisphere (T2 FLAIR [fluid attenuated inversion recovery], June 11, 2018). (B) Postsurgery MRI highlighting the complete tumor resection (T2 FLAIR, June 13, 2018). (C) Monitoring MRI, T2 FLAIR, September 2019. (D) Monitoring MRI, T2 FLAIR sequence in posterior fossa, performed in December 2021. (E) Monitoring MRI, T2 sequence of the frontal lobe, showing no lesion in December 2021. (F) MRI T2 FLAIR performed in March 2022 showing the second primary brain tumor (dpHGG) in the frontal lobe.

**Figure S2.** Patient‐derived xenotransplantation (PDX) of the dpHGG (diffuse pediatric‐type high‐grade glioma) from the CMMRD (constitutional mismatch repair deficiency) patient. (A) Subcutaneous dpHGG derived from CMMRD with biallelic *MSH6* variant. (B) H&E (hematoxylin and eosin) stain of the dpHGG (PDX) from CMMRD patient.

**Figure S3.** SBS (single base substitution) reconstruction plot for the medulloblastoma, dpHGG (diffuse pediatric‐type high‐grade glioma), and PDX (dpHGG). This plot reports the contribution of each identified mutational signature, total mutation number, cosine similarity, and other statistics.

**Figure S4.** Indel (ID) mutational profile for the medulloblastoma, dpHGG (diffuse pediatric‐type high‐grade glioma), and PDX (dpHGG).

**Figure S5.** Single base substitution (SBS) signature contribution, SBS, and indel mutation for CMMRD (constitutional mismatch repair deficiency)–associated brain tumors. (A) Heat map showing the contribution of each SBS and ID signature to individual samples. (B) Bar plots indicate the total number of SBS and (C) indel (ID) mutations. Cosine similarities for SBS were 0.997, 0.991, and 0.940 for dpHGG, dpHGG PDX, and medulloblastoma, respectively. Cosine similarities for ID were 0.995, 0.961, and 0.936 for dpHGG, dpHGG PDX, and medulloblastoma, respectively.

**Table S1.** Percentage of target regions coverage in the Medulloblastomas, dpHGG, PDX (dpHGG) and normal (Blood) DNA from the patient with CMMRD.

**Table S2.** Total and tumor‐specific variants identified by whole exome sequencing of the CMMRD‐derived tumor samples.

**Table S3.** List of the 152 CNS tumors associated genes.

**Table S4.** Microsatellite Instability (MSI) tests using PCR (custom assay using 6 markers), Whole exome sequencing (WES) MSI based on 117 markers (Sophia Genetics), and MSI based on WES (Dragen Illumina) in primary brain tumors and in the PDX derived from CMMRD.

**Table S5.** SBS and ID mutational signature information.

## Data Availability

The datasets used and analyzed during the current study are available from the corresponding author upon reasonable request. The exome data are available at The European Genome‐phenome Archive (EGA) https://ega‐archive.org/, ID EGAS50000000351.
