# Supplementary material for: Establishment and molecular profiling of a PDX model of a metachronous brain tumor in a patient with constitutional mismatch repair deficiency with biallelic MSH6 variant
Source: Animal Model Exp Med. 2025 Aug 29;8(11):1971–82. doi: 10.1002/ame2.70069 (PMC12746185; doi:10.1002/ame2.70069)
Supplement: Supplementary file 1 — Data S1. Detailed description of the methods. [file AME2-8-1971-s003.docx]

**Supplementary Material 1**

Clinical data collection

The data from the patient, such as magnetic resonance images, clinical and pathological exam results, and patients' evolution history, were collected in medical reports in the hospital information system (TASY, Philips Medical Systems, Netherlands) at the Pediatric Department of Barretos Cancer Hospital (BCH), São Paulo, Brazil. The family history and the germline mutation analysis were collected in the Oncogenetics Department of BCH ^1^. The pathology department and the biobank ^2^ from BCH provided the FFPE and frozen tumor tissues (medulloblastoma and diffuse pediatric-type high-grade glioma) and blood samples, respectively, for the molecular characterization.

Histopathology and immunohistochemistry

Histopathological analysis was conducted by two experienced neuropathologists from the Department of Molecular Pathology at Barretos Cancer Hospital. Diagnoses were established based on cell morphology, molecular analysis, and immunohistochemistry (IHC).

For the IHC of mismatch repair proteins (MLH1, MSH2, MSH6, and PMS2), 3 μm sections of FFPE tissue blocks were prepared and subjected to immunohistochemical staining using the Dako EnVision™ FLEX detection system Kit and Autostainer Link 48 equipment (Dako, Glostrup, Denmark). Antigen retrieval was conducted at 97°C for 20 minutes (pH 9.0). Endogenous peroxidases were blocked using EnVision™ FLEX Peroxidase-Blocking Reagent (Dako, Glostrup, Denmark), following the manufacturer’s protocol^5^. The primary antibodies included FLEX monoclonal mouse anti-MutL protein homolog 1 (MLH1) (clone ES05, ref IS079), FLEX monoclonal mouse anti-MutS protein homolog 2 (MSH2) (clone FE11, ref IR085), FLEX monoclonal rabbit anti-postmeiotic segregation increased 2 (PMS2) (clone EP51, ref IR087), and FLEX monoclonal rabbit anti-MutS protein homolog 6 (MSH6) (clone EP49, ref IR086) (Dako, Glostrup, Denmark). Immunostaining visualization was achieved using the DAB solution, and hematoxylin was applied for counterstaining^5^.

The IHC analysis of PD-L1 was conducted on CMMRD samples using the Dako EnVision FLEX and HRP-polymer kit. The slides underwent deparaffinization rehydration and antigen retrieval (Dako EnVision FLEX Target Retrieval pH6). Staining was executed in the Dako Automated Link 48, and the PD-L1 antibody (22C3 pharmDx) was prepared according to the manufacturer's guidelines and aligned with previous studies. Finally, the slides were counterstained with hematoxylin. The intensity of staining was assessed as follows: 0 (negative), 1+ (weak), 2+ (moderate), or 3+ (strong), as previously described^6^. An experienced neuropathologist evaluated the results.

Molecular analysis

Nucleic acid IsolationTumor DNA was extracted using the QIAsymphony DNA Mini Kit (Qiagen) on the automated QIAsymphony platform (Qiagen), while DNA from peripheral blood was obtained using the QIAamp DNA Blood Mini QIAcube Kit (Qiagen), in accordance with the manufacturer's instructions. DNA quality was assessed with Nanodrop (Thermo Fisher Scientific) and quantified using Qubit Fluorometric Quantitation (Thermo Fisher Scientific), following the manufacturer's guidelines.

RNA was isolated using the RNeasy Mini Kit (Qiagen), and RNA quantification was carried out using the Qubit 2.0 Fluorometer (RNA HS Assay Kit, Thermo Fisher Scientific) in accordance with the manufacturer's guidelines^4^.

Medulloblastoma Molecular Classification

Following RNA isolation from tumor FFPE, the molecular classification of primary medulloblastoma was performed based on the mRNA 22-gene signature by nCounter as previously reported ^7,8^. Once classified as an SHH-activated subgroup, the analysis of somatic variants on *TP53* was performed using the Trusight Tumor 15 NGS panel with MiSeq System according to manufacturer’s instructions (Illumina, US) as reported ^9^.

Sanger sequencing of *IDH1, IDH2* and *H3F3A*

The target mutation analysis (hotspots) of the *IDH1* gene (exon 4, codon 132), IDH2 (exon 4, codons 140 and 172) and H3F3A (exon 2, codons 27 and 34) was based on PCR followed by direct sequencing. The region of interest of the IDH1 gene was amplified by PCR using the primers 5’-CGGTCTTCAGAGAAGCCATT-3’ (forward) and 5’-CACATTATTGCCAACATGAC-3’ (reverse); for IDH2, with primers 5’-CCACTATTATCTCTGTCCTC-3’ (forward) and 5’-GCTAGGCGAGGAGCTCCAGT-3’ (reverse); for H3F3A, with primers 5’-CATGGCTCGTACAAAGCAGA-3’ (forward) and 5'-CAAGAGAGACTTTGTCCCATTTTT-3’ (reverse). Amplification products were confirmed by gel electrophoresis. Sequencing was performed using the BigDye® Terminator v3.1 Cycle Sequencing Kit (Applied Biosystems, USA) and evaluated with the ABI PRISM 3500 xL Genetic Analyzer® (Applied Biosystems, USA).

*TP53* and *MSH6* germline sequencing

Genomic DNA was isolated from peripheral blood samples using the QIAamp DNA Blood MiniKit (Qiagen) following the manufacturer’s instructions. DNA quantification was determined using the Qubit dsDNA Broad range Assay Kit (Thermo Fisher Scientific—United States)^10^.

The germline variants on TP53 gene (NM_000546.6) was evaluated by NGS using the Custom Hereditary Rare Cancer Solution kit (Sophia Genetics, Switzerland), which includes the genes APC, BRCA2, CEBPA, DICER1, GATA2, SMARCB1, MEN1, NF1, NF2, PALB2, PTCH1, PTEN, RB1, RET, RUNX1, SUFU, TP53, TSC1, TSC2, and VHL, according to the manufacturer’s protocol and as reported.

The analysis of germline variants on MSH6 (NM_000179.2) was also evaluated by NGS. Library construction was carried out with the Custom Hereditary Cancer Solution (CHCS) kit (Sophia Genetics, Switzerland) according to the manufacturer’s protocol. Briefly, DNA fragments were generated using an enzymatic fragmentation step. The three subsequent enzymatic steps, end-repair, A-tailing, and ligation to Illumina adapters, were performed to produce NGS libraries. Capture-based target enrichment was carried out on the pooled libraries. The final pool of libraries was quantitated using Qubit dsDNA HS fluorometric assays (Life Technologies, USA). Quality control of fragment size was assessed using DNA Screen Tape analysis (4150 Tape Station system, Agilent). Sequencing was achieved with the final library concentration of 10 pM onto a 600-cycle format V3 flow-cell via the Illumina MiSeq platform (Illumina, San Diego, CA, USA).

Data analysis was performed to detect Single Nucleotide Variants (SNVs), and insertions/deletions (indels). Sequencing FASTQ data was analyzed using the Sophia DDM® platform (Sophia Genetics, Switzerland). The classification of each genomic variant into five different categories: benign (B), likely benign (LB), variant of uncertain significance (VUS), likely pathogenic (LP), and pathogenic (P) was performed according to the American College of Medical Genetics and Genomics (ACMG) guidelines.

DNA methylation profile

The methylation array was performed using 500ng of DNA extracted from cryopreserved tumor samples. In the present study, we used the methylation EPIC Bead Chip Infinium microarray v2.0 kit (935k) (Illumina, San Diego, CA, USA), which is a genome wide methylation screening tool that covers over 935k CpG sites, annotated in the human genome version GRCh38/h38. The experiments were performed in the Next Seq 550 System (Illumina, San Diego, CA, USA) at the laboratory of Molecular Diagnosis from Barretos Cancer Hospital, Brazil, according to manufacturer’s recommendations.

The analysis of the copy number variation (CNV) profiles and CNV plot based on raw DNA methylation array data, was performed using the conumee 2.0 tool (https://doi.org/10.1093/bioinformatics/btae029) in the version integrated into the DKFZ/Heidelberg tumor classifier platform v12.8 from the German Cancer Research Center platform as implemented in the classifier package (https://doi.org/10.1038/nature26000). The approach provides de visualization of chromosomes 1 to 22, X and Y. Positive deviations are depicted as gains/amplifications, while negative ones signify losses from the baseline. Notable emphasis is placed on 29 gene regions relevant to brain tumors, facilitating easier evaluation. The reports including the molecular classification of CMMRD derived brain tumors, were also generated using DKFZ/Heidelberg classifier v12.8 (available at [www.molecularneuropathology.org/](http://www.molecularneuropathology.org/)).

Whole exome sequencing (WES) and analysis

DNA isolation was performed on frozen tumor tissue, with a minimum of 60% tumor cells and no more than 20% necrosis, and a peripheral blood sample was obtained from the patient and stored at the Biobank from Barretos Cancer Hospital. The automated QIAsymphony platform (QIAGEN) was employed to isolate tumor DNA, utilizing the QIAsymphony DNA mini-Kit (Qiagen, Valencia, CA). Concurrently, peripheral blood DNA extraction utilized the QIAamp DNA Blood Mini QIAcube Kit (Qiagen, Valencia, CA). DNA quality was assessed using Nanodrop (Thermo Scientific, Waltham, MA, USA), and quantification was performed using Qubit 2.0 Fluorometer (Thermo Fisher Scientific, Waltham, MA USA), following the respective manufacturers' guidelines.

The SOPHiA Genetics Facility conducted whole-exome sequencing on the Illumina NovaSeq 6000 System platform, employing the SOPHiA Whole Exome Solution kit, with 200 ng of DNA utilized for both somatic and germline analyses. Subsequently, the sequencing fastq files were analyzed at the platform SOPHiA DDM version 4, SOPHiA Genetics, based on the GRCh38/hg19.

Microsatellite instability (MSI)

Distinct approaches evaluated MSI. Initially, MSI was assessed on DNA isolation from FFPE of medulloblastoma, dpHGG, and PDX, using a multiplex polymerase chain reaction (PCR) Multiplex Kit (Qiagen, Venlo, The Netherlands) involving six quasi-monomorphic mononucleotide repeat markers: BAT-25, BAT-26, NR-21, NR-24, NR-27, and HSP110 was reported ^11^.

The Sophia Genetics MSI detection algorithm from the whole exome sequencing (WES) is based on examining variations in the length of a subset of 117 sequences of repeated nucleotides (homopolymers), each consisting of at least 12 nucleotides, within designated regions of the CSTS_N_v2 panel. The choice of these homopolymers was determined using statistical analysis and a custom-built algorithm developed by the researchers to identify the most pertinent genetic loci for MSI detection.

Next, we evaluated the MSI from WES using the Illumina’s BaseSpace™ Sequence Hub. Sequence reads were aligned to the GRCh38 reference genome (UCSC hg38 Alt-Masked v3) utilizing the DRAGEN Somatic App v4.2.4 (Illumina, Inc., California, USA). In the MSI Biomarkers module with enabled tumor-normal mode (Illumina DRAGEN Bio-IT Platform v4.2 documentation, https://support-docs.illumina.com/SW/dragen_v42/Content/SW/DRAGEN/Biomarkers_MSI.htm?Highlight=msi), the number of aligned reads from tumor and normal samples was counted separately and the Jensen-Shannon Distance was applied in the accessible sites to estimate how the distribution of DNA fragments at a microsatellite site differs between tumor and normal samples. As a result, unstable sites are significantly shifted between tumor and normal. For solid tumor samples, the percentage of unstable sites is equal to or higher than 20%, which indicates microsatellite instability. Additionally, tumor mutational burden (TMB) was also enabled and evaluated with the DRAGEN Somatic App.

Mutational Signatures

A local python Sig Profiler Assignment version 0.1.9 and the web server (<https://cancer.sanger.ac.uk/signatures/assignment/>) were used in the refitting approach to assign the known mutational signatures for single base substitution (SBS), doublet base substitution (DBS) and small insertion and deletion (ID) using the version v3.4 of the COSMIC mutational signatures catalog. The assignment evaluation obtained cosine similarity greater than 0.90 for SBS and IDS mutational signatures and the value to DBS ones less than 0,757 (data not shown).

The immune checkpoint expression profile

We used the nCounter Human PanCancer Immune Profiling Panel to analyze the immune-related mRNA, with 730 immune oncology-related targets and 40 internal reference controls (NanoString Technologies) as reported^4^. The nSolver Analysis Software v4.0 by NanoString Technologies® was employed to evaluate quality control parameters, encompassing binding density, limit of detection, and positive controls. Following this, the Advanced Analysis module (NanoString Technologies®) was utilized to normalize raw data using housekeeping genes with lower variation, as determined by the geNorm algorithm. This module also facilitated differential expression analysis and the computation of immune-oncology-related scores. The immune checkpoint-related genes' heatmaps were generated using GraphPad Prism 9 based on normalized mRNA data.

Personalized panel targeting genes associated with Central Nervous System (CNS) tumors

To evaluate single-nucleotide variants (SNVs), small insertions/deletions (InDels) variants associated with central nervous system tumors, we constructed a personalized virtual panel containing 152 CNS tumor-associated genes listed in **Supplementary Table 1**. Our panel includes all relevant genes described on the WHO 2021 classifications for CNS tumors ^12^and 130 genes included in a large study involving pediatric neuro-oncology multiomics from Heidelberg ^13^.

References

1 Palmero EI, Galvão HCR, Fernandes GC, De Paula AE, Oliveira JC, Souza CP *et al.* Oncogenetics service and the Brazilian public health system: the experience of a reference Cancer Hospital. *Genet Mol Biol* 2016; **39**: 168–177.

2 Neuber AC, Tostes CH, Ribeiro AG, Marczynski GT, Komoto TT, Rogeri CD *et al.* The biobank of barretos cancer hospital: 14 years of experience in cancer research. *Cell Tissue Bank* 2022; **23**: 271–284.

3 Teixeira SA, Luzzi M de C, Martin ACBM, Duarte TT, Leal M de O, Teixeira GR *et al.* The Barretos Cancer Hospital Animal Facility: Implementation and Results of a Dedicated Platform for Preclinical Oncology Models. *Vet Sci* 2022; **9**. doi:10.3390/VETSCI9110636.

4 Moreno DA, Silva LS da, Gomes I, Leal LF, Berardinelli GN, Gonçalves GM *et al.* Cancer immune profiling unveils biomarkers, immunological pathways, and cell type score associated with glioblastoma patients’ survival. *Ther Adv Med Oncol* 2022; **14**: 175883592211276.

5 Berardinelli GN, Durães R, Mafra da Costa A, Bragagnoli A, Antônio de Oliveira M, Pereira R *et al.* Association of microsatellite instability (MSI) status with the 5-year outcome and genetic ancestry in a large Brazilian cohort of colorectal cancer. *Eur J Hum Genet* 2022; **30**: 824–832.

6 Marques RF, Moreno DA, da Silva L, Leal LF, de Paula FE, Santana I *et al.* Digital expression profile of immune checkpoint genes in medulloblastomas identifies CD24 and CD276 as putative immunotherapy targets. *Front Immunol* 2023; **14**. doi:10.3389/FIMMU.2023.1062856.

7 Northcott PA, Shih DJH, Remke M, Cho YJ, Kool M, Hawkins C *et al.* Rapid, reliable, and reproducible molecular sub-grouping of clinical medulloblastoma samples. *Acta Neuropathol* 2012; **123**: 615–626.

8 Leal LF, Evangelista AF, de Paula FE, Caravina Almeida G, Carloni AC, Saggioro F *et al.* Reproducibility of the NanoString 22-gene molecular subgroup assay for improved prognostic prediction of medulloblastoma. *Neuropathology* 2018; **38**: 475–483.

9 Barateiro LGRP, de Oliveira Cavagna R, dos Reis MB, de Paula FE, Teixeira GR, Moreno DA *et al.* Somatic mutational profiling and clinical impact of driver genes in Latin-Iberian medulloblastomas: Towards precision medicine. *Neuropathology* 2024. doi:10.1111/NEUP.12979.

10 da Silva LS, Mançano BM, de Paula FE, dos Reis MB, de Almeida GC, Matsushita M *et al.* Expression of GNAS, TP53, and PTEN Improves the Patient Prognostication in Sonic Hedgehog (SHH) Medulloblastoma Subgroup. *J Mol Diagn* 2020; **22**: 957–966.

11 Berardinelli GN, Scapulatempo-Neto C, Durães R, de Oliveira MA, Guimarães D, Reis RM. Advantage of HSP110 (T17) marker inclusion for microsatellite instability (MSI) detection in colorectal cancer patients. *Oncotarget* 2018; **9**: 28691.

12 Louis DN, Perry A, Wesseling P, Brat DJ, Cree IA, Figarella-Branger D, Hawkins C, Ng HK, Pfister SM, Reifenberger G, Soffietti R von DA, Ellison D. The 2021 WHO Classification of Tumors of the Central Nervous System: a summary. *Neuro Oncol* 2021; **23**: 1231–1251.

13 Sturm D, Capper D, Andreiuolo F, Gessi M, Kölsche C, Reinhardt A *et al.* Multiomic neuropathology improves diagnostic accuracy in pediatric neuro-oncology. *Nature Medicine 2023 29:4* 2023; **29**: 917–926.
