# Supplementary figures and images for: Establishment and molecular profiling of a PDX model of a metachronous brain tumor in a patient with constitutional mismatch repair deficiency with biallelic MSH6 variant
Source: Animal Model Exp Med. 2025 Aug 29;8(11):1971–82. doi: 10.1002/ame2.70069 (PMC12746185; doi:10.1002/ame2.70069)

**A**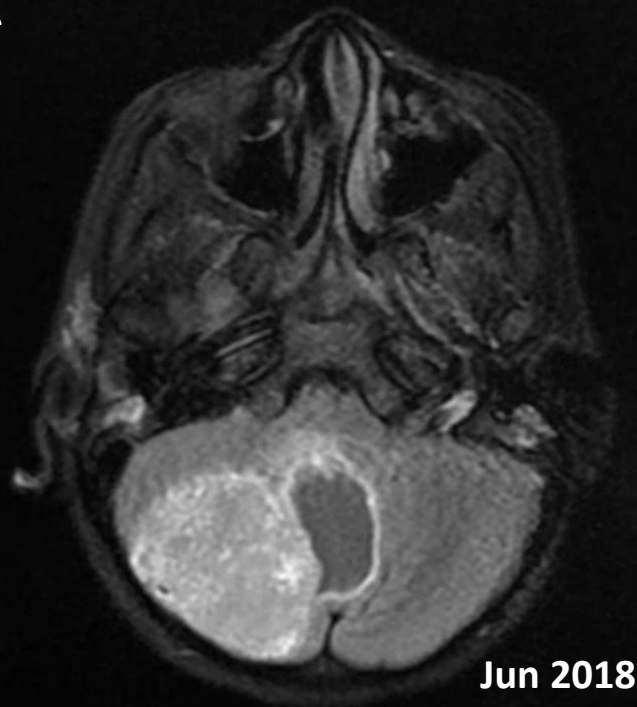**B**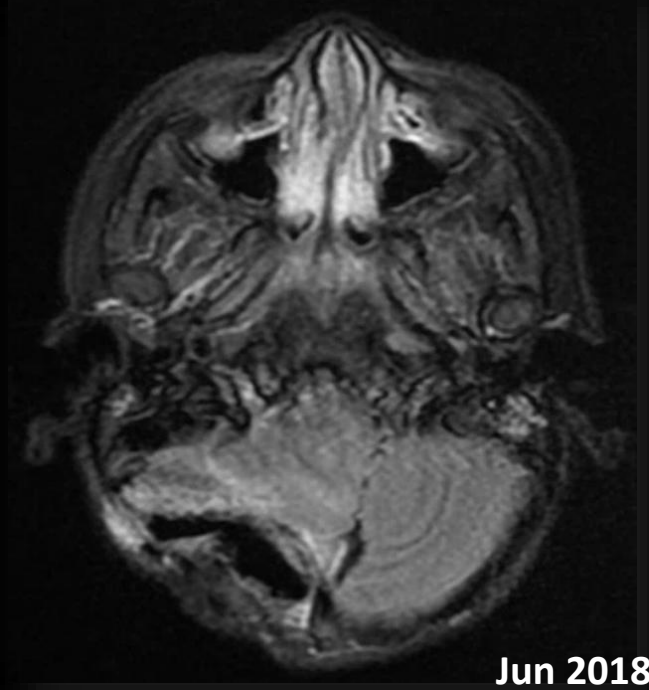**C**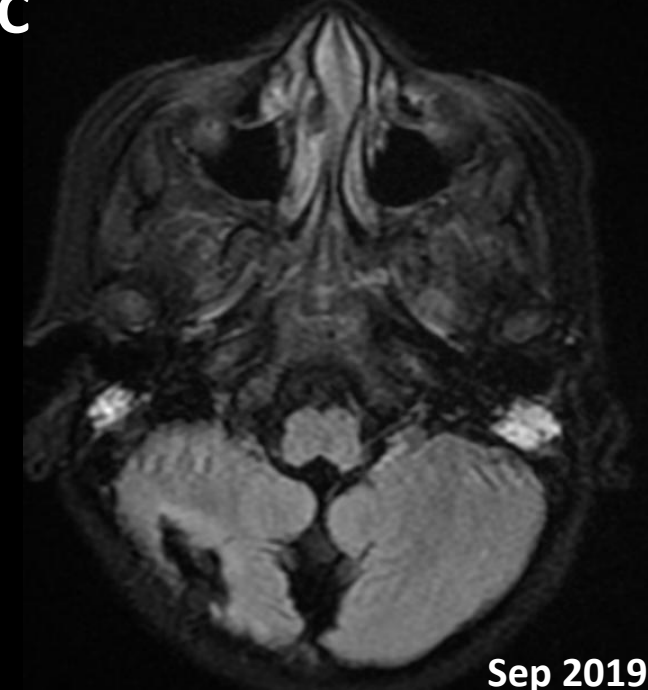**D**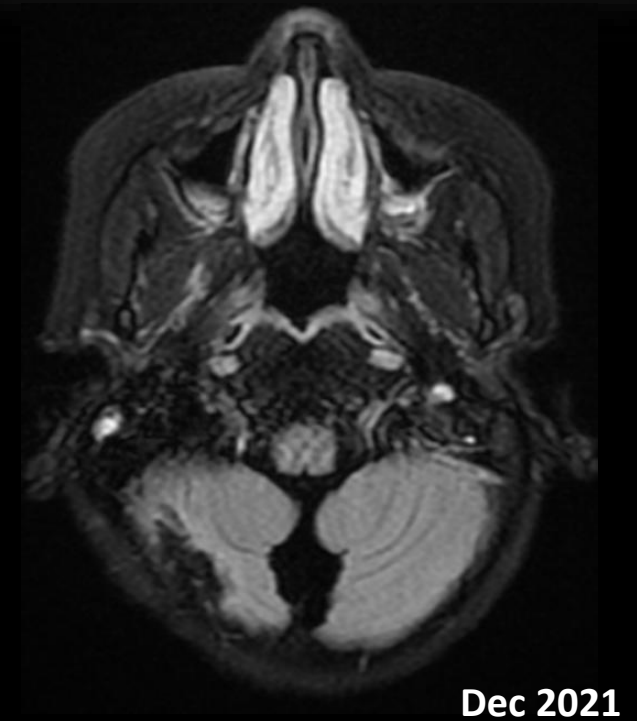**E**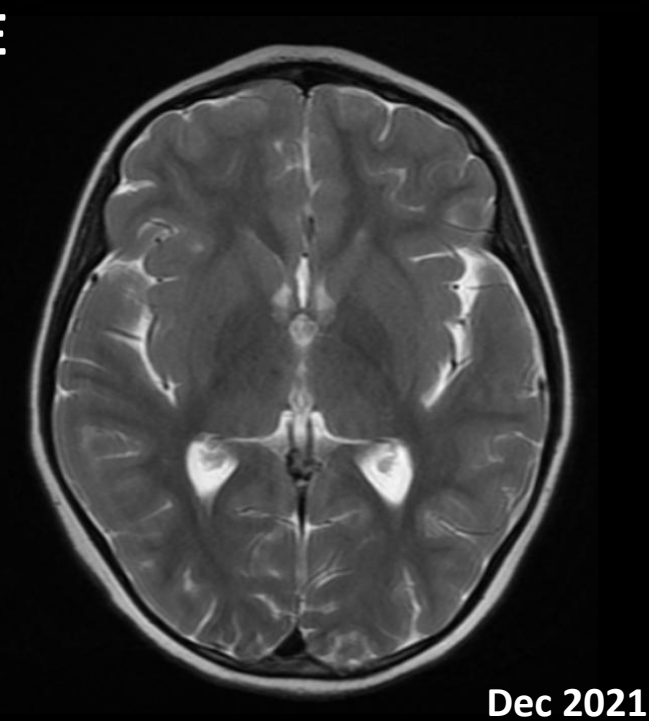**F**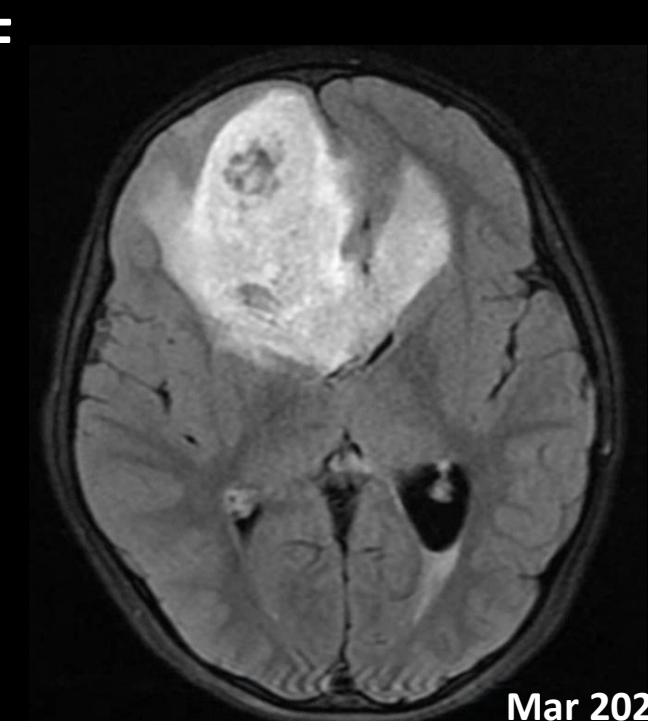

Supplement: Supplementary file 2 — Figure S1. Brain magnetic resonance imaging (MRI) monitoring the clinical evaluation of the CMMRD (constitutional mismatch repair deficiency) patient. (A) Diagnosis of medulloblastoma in the left cerebellar hemisphere (T2 FLAIR [fluid attenuated inversion recovery], June 11, 2018). (B) Postsurgery MRI highlighting the complete tumor resection (T2 FLAIR, June 13, 2018). (C) Monitoring MRI, T2 FLAIR, September 2019. (D) Monitoring MRI, T2 FLAIR sequence in posterior fossa, performed in December 2021. (E) Monitoring MRI, T2 sequence of the frontal lobe, showing no lesion in December 2021. (F) MRI T2 FLAIR performed in March 2022 showing the second primary brain tumor (dpHGG) in the frontal lobe. [file AME2-8-1971-s004.pdf]

**A**

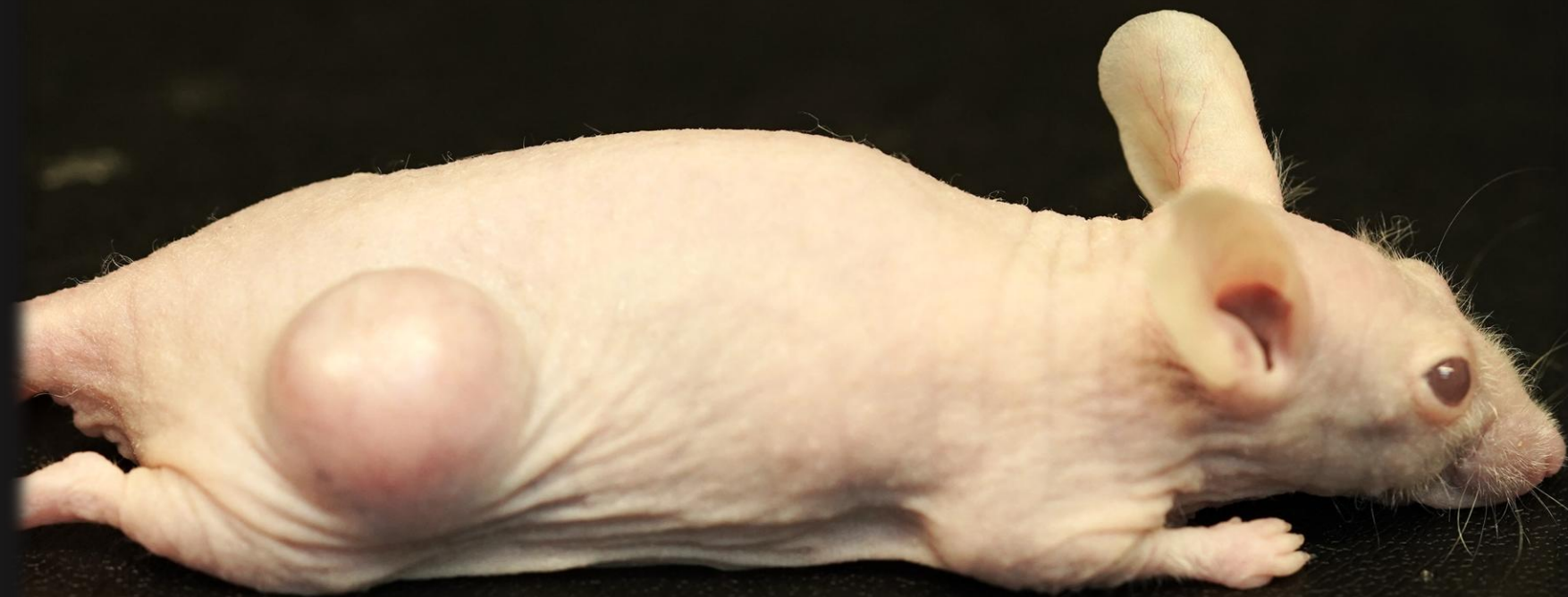

**PDX (dpHGG)**

**B**

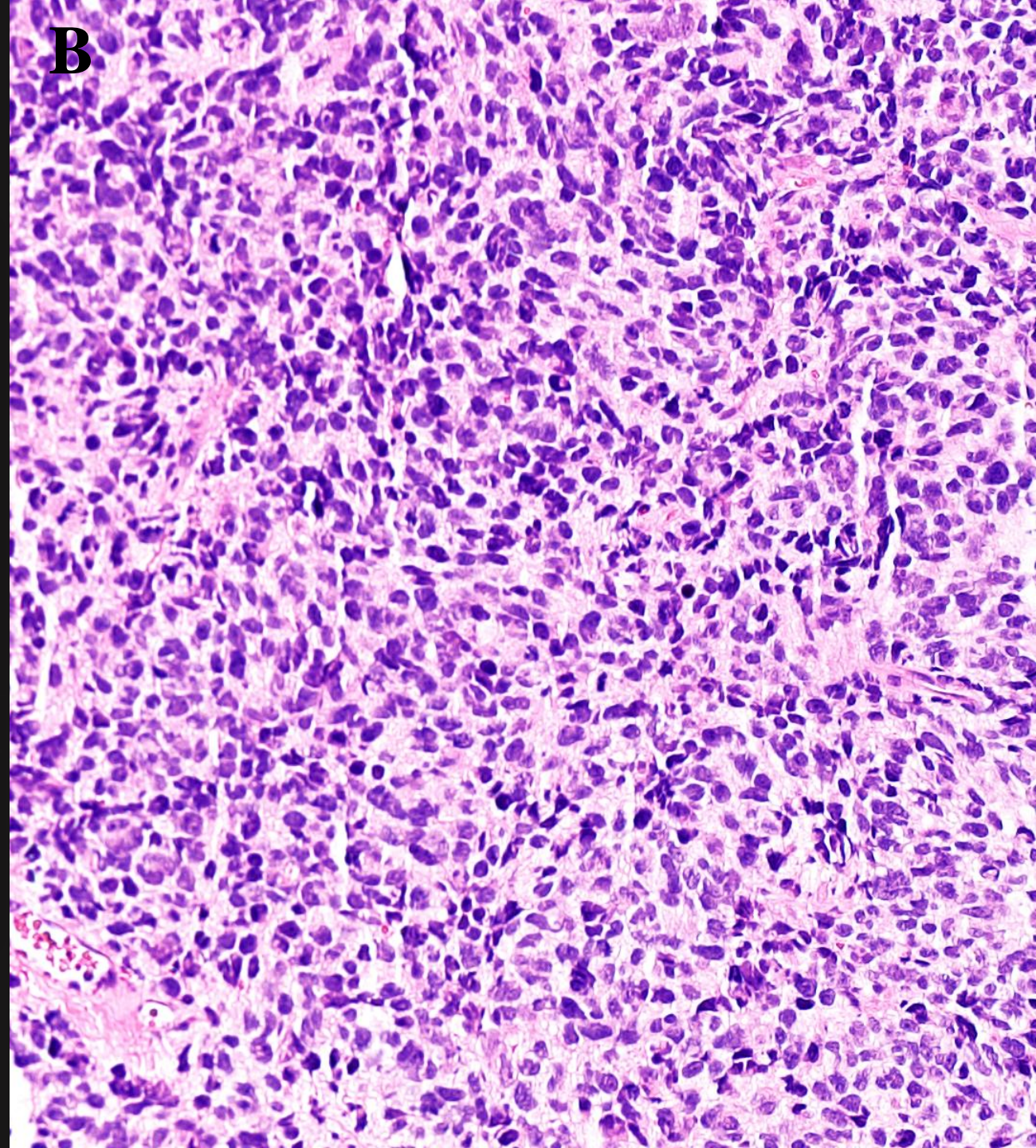

Supplement: Supplementary file 3 — Figure S2. Patient‐derived xenotransplantation (PDX) of the dpHGG (diffuse pediatric‐type high‐grade glioma) from the CMMRD (constitutional mismatch repair deficiency) patient. (A) Subcutaneous dpHGG derived from CMMRD with biallelic MSH6 variant. (B) H&E (hematoxylin and eosin) stain of the dpHGG (PDX) from CMMRD patient. [file AME2-8-1971-s011.pdf]

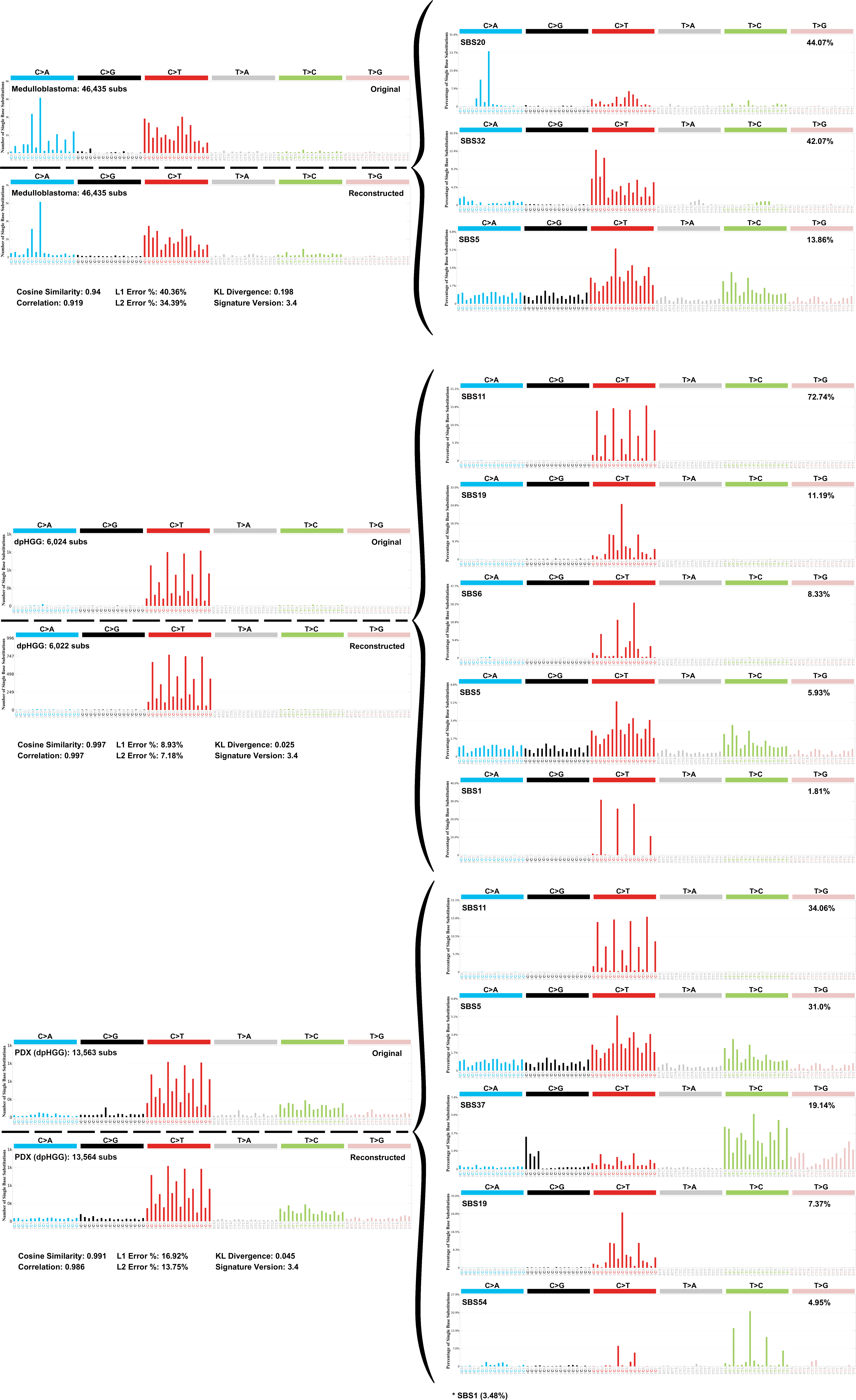

Supplement: Supplementary file 4 — Figure S3. SBS (single base substitution) reconstruction plot for the medulloblastoma, dpHGG (diffuse pediatric‐type high‐grade glioma), and PDX (dpHGG). This plot reports the contribution of each identified mutational signature, total mutation number, cosine similarity, and other statistics. [file AME2-8-1971-s005.png]

## Slide 1
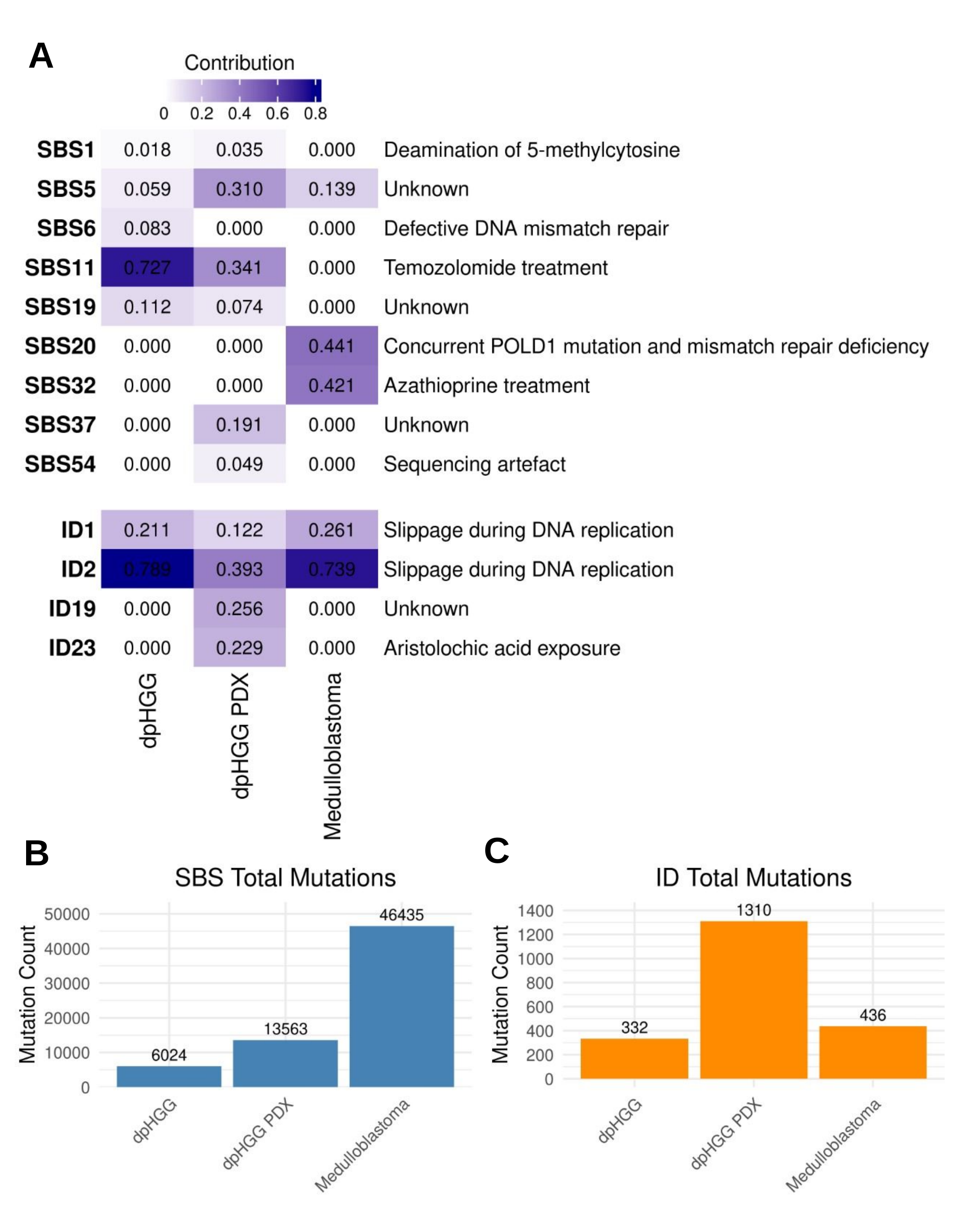

A
C
B

Supplement: Supplementary file 6 — Figure S5. Single base substitution (SBS) signature contribution, SBS, and indel mutation for CMMRD (constitutional mismatch repair deficiency)–associated brain tumors. (A) Heat map showing the contribution of each SBS and ID signature to individual samples. (B) Bar plots indicate the total number of SBS and (C) indel (ID) mutations. Cosine similarities for SBS were 0.997, 0.991, and 0.940 for dpHGG, dpHGG PDX, and medulloblastoma, respectively. Cosine similarities for ID were 0.995, 0.961, and 0.936 for dpHGG, dpHGG PDX, and medulloblastoma, respectively. [file AME2-8-1971-s008.pptx]
