# Supplementary material for: Establishment and molecular profiling of a PDX model of a metachronous brain tumor in a patient with constitutional mismatch repair deficiency with biallelic MSH6 variant
Source: Animal Model Exp Med. 2025 Aug 29;8(11):1971–82. doi: 10.1002/ame2.70069 (PMC12746185; doi:10.1002/ame2.70069)
Supplement: Supplementary file 7 — Table S1. Percentage of target regions coverage in the Medulloblastomas, dpHGG, PDX (dpHGG) and normal (Blood) DNA from the patient with CMMRD. [file AME2-8-1971-s009.docx]

**Supplementary Table 1.**  Percentage of target regions coverage in the Medulloblastomas, dpHGG, PDX (dpHGG) and normal (Blood) DNA from the patient with CMMRD.

| **Sample ID** | **25×** | **50×** | **100×** | **200×** | **500×** | **1000×** | **Coverage 10% quantile** | **Coverage Heterogeneity** |
| --- | --- | --- | --- | --- | --- | --- | --- | --- |
| Blood E1819778 | 98.20% | 96.76% | 81.64% | 34.93% | 0.72% | 0.06% | 79× | 2.12% |
| Medulloblastoma | 98.30% | 98.18% | 97.92% | 95.22% | 52.89% | 6.40% | 263× | 2.17% |
| Blood E227837 | 87.64% | 86.17% | 77.84% | 22.52% | 0.05% | 0.00% | 1× | 12.71% |
| dpHGG E227837 | 89.14% | 88.46% | 87.60% | 86.51% | 77.36% | 18.99% | 8× | 13.03% |
| PDX E227837 | 89.02% | 88.32% | 87.44% | 86.29% | 73.81% | 12.81% | 7× | 13.09% |
