# Supplementary material for: Establishment and molecular profiling of a PDX model of a metachronous brain tumor in a patient with constitutional mismatch repair deficiency with biallelic MSH6 variant
Source: Animal Model Exp Med. 2025 Aug 29;8(11):1971–82. doi: 10.1002/ame2.70069 (PMC12746185; doi:10.1002/ame2.70069)
Supplement: Supplementary file 8 — Table S2. Total and tumor‐specific variants identified by whole exome sequencing of the CMMRD‐derived tumor samples. [file AME2-8-1971-s010.docx]

**Supplementary Table 2.** Total and tumor-specific variants identified by whole exome sequencing of the CMMRD-derived tumor samples.

|  | **Medulloblastoma** | | **dpHGG** | | **PDX (dpHGG)** | |
| --- | --- | --- | --- | --- | --- | --- |
| Number of Variants | **Total** | **Tumor Specific** | **Total** | **Tumor Specific** | **Total** | **Tumor Specific** |
| Sophia Total Variants | 70195 | 35941 | 30874 | 2273 | 123198 | 87164 |
| (A) Pathogenic | 3019 | 2413 | 540 | 107 | 1049 | 396 |
| (B) Likely Pathogenic | 22368 | 19693 | 4183 | 1306 | 17008 | 12717 |
| (C) Uncertain Significant | 38840 | 13832 | 20694 | 859 | 99723 | 74004 |
| (D) Likely Benign | 5968 | 3 | 5457 | 1 | 5418 | 7 |
