# Supplementary material for: Establishment and molecular profiling of a PDX model of a metachronous brain tumor in a patient with constitutional mismatch repair deficiency with biallelic MSH6 variant
Source: Animal Model Exp Med. 2025 Aug 29;8(11):1971–82. doi: 10.1002/ame2.70069 (PMC12746185; doi:10.1002/ame2.70069)
Supplement: Supplementary file 9 — Table S3. List of the 152 CNS tumors associated genes. [file AME2-8-1971-s002.docx]

**Supplementary Table 3.** List of the 152 CNS tumors associated genes.

| **Central Nervous System (CNS) tumors associated genes** | | | | | | | |
| --- | --- | --- | --- | --- | --- | --- | --- |
| *ABL1* | *C11ORF95* | *EGFR* | *HIST1H3C* | *MDM2* | *NOTCH1* | *PRKAR1A* | *SMO* |
| *ACVR1* | *CCND1* | *ERCC1* | *HNF1A* | *MDM4* | *NOTCH2* | *PRKCA* | *STAG2* |
| *AKT1* | *CCND2* | *EZH2* | *HRAS* | *MET* | *NRAS* | *PTCH1* | *SUFU* |
| *AKT2* | *CDH1* | *FBXW7* | *IDH1* | *MGMT* | *NTRK1* | *PTCH2* | *TBR1* |
| *AKT3* | *CDK4* | *FGFR1* | *IDH2* | *MLH1* | *NTRK2* | *PTEN* | *TCF4* |
| *ALK* | *CDK6* | *FGFR2* | *IDO2* | *MLL2* | *NTRK3* | *PTPN11* | *TERT* |
| *APC* | *CDKN2A* | *FGFR3* | *JAK2* | *MN1* | *OLIG2* | *Rad50* | *TP53* |
| *ARID1A* | *CDKN2B* | *FGFR4* | *JAK3* | *MPL* | *PALB2* | *RAF1* | *TRAF7* |
| *ARID1B* | *CDKN2C* | *FLT3* | *KDM6A* | *MRE11A* | *PCDH8* | *RB1* | *TSC1* |
| *ARID2* | *CHEK2* | *FOXO3* | *KDR* | *MSH2* | *PDGFRA* | *RELA* | *TSC2* |
| *ATM* | *CIC* | *FUBP1* | *KIAA0182* | *MSH6* | *PIK3C2G* | *RET* | *VHL* |
| *ATR* | *CREBBP* | *GABRA6* | *KIAA1549* | *MYB* | *PIK3CA* | *ROS1* | *YAP1* |
| *ATRX* | *CSF1R* | *GLI2* | *KIT* | *MYBL1* | *PIK3R1* | *SETD2* |  |
| *BAP1* | *CTNNB1* | *GNA11* | *KLF4* | *MYC* | *PLAUR* | *SMAD4* |  |
| *BCOR* | *CYSLTR2* | *GNAQ* | *KLK1* | *MYCN* | *PLCB4* | *SMARCA2* |  |
| *BRAF* | *D2HGDH* | *GNAS* | *KRAS* | *MYL1* | *PMS2* | *SMARCA4* |  |
| *BRCA1* | *DAXX* | *H2AFX* | *LDB1* | *NBN* | *POLD1* | *SMARCB1* |  |
| *BRCA2* | *DDX3X* | *H3F3A* | *LZTR1* | *NDRG2* | *POLE* | *SMARCD1* |  |
| *BRPF1* | *DICER1* | *HDAC2* | *MAD2L2* | *NF1* | *PPM1D* | *SMARCD2* |  |
| *BRPF3* | *E2F2* | *HIST1H3B* | *MAML2* | *NF2* | *PRDM6* | *SMARCE1* |  |
