# Supplementary material for: Establishment and molecular profiling of a PDX model of a metachronous brain tumor in a patient with constitutional mismatch repair deficiency with biallelic MSH6 variant
Source: Animal Model Exp Med. 2025 Aug 29;8(11):1971–82. doi: 10.1002/ame2.70069 (PMC12746185; doi:10.1002/ame2.70069)
Supplement: Supplementary file 10 — Table S4. Microsatellite Instability (MSI) tests using PCR (custom assay using 6 markers), Whole exome sequencing (WES) MSI based on 117 markers (Sophia Genetics), and MSI based on WES (Dragen Illumina) in primary brain tumors and in the PDX derived from CMMRD. [file AME2-8-1971-s001.docx]

**Supplementary** **Table 4.** Microsatellite Instability (MSI) tests using PCR (custom assay using 6 markers), Whole exome sequencing (WES) MSI based on 117 markers (Sophia Genetics), and MSI based on WES (Dragen Illumina) in primary brain tumors and in the PDX derived from CMMRD.

|  | PCR (6 markers)^23^ | Sophia Genetics based on 117 markers (MSI score cutoff > 0.005)^17^ | Dragen Illumina  WES (MSI = unstable sites > 20 %) |
| --- | --- | --- | --- |
| Medulloblastoma | MSS | Not performed | MSI (21.94%) |
| HGG | MSS | MSS (0.003) | MSS (11.3%) |
| PDX | MSS | MSI-LC (0.005) | MSS (14.75%) |

PCR: Polymerase Chain Reaction; MSS: Microsatellite Stability; MSI: Microsatellite instability; WES: Whole Exome Sequencing; HGG: High Grade Glioma; PDX: Patient Derived Xenotransplantation; LC: Low Confidence (Threshold limit).
