# Supplementary material for: Establishment and molecular profiling of a PDX model of a metachronous brain tumor in a patient with constitutional mismatch repair deficiency with biallelic MSH6 variant
Source: Animal Model Exp Med. 2025 Aug 29;8(11):1971–82. doi: 10.1002/ame2.70069 (PMC12746185; doi:10.1002/ame2.70069)
Supplement: Supplementary file 11 — Table S5. SBS and ID mutational signature information. [file AME2-8-1971-s007.docx]

**Supplementary Table 5.** SBS and ID mutational signature information.

| Signature | Proposed Etiology | dpHGG | dpHGG PDX | Medulloblastoma |
| --- | --- | --- | --- | --- |
| SBS | | | | |
| SBS1 | Deamination of 5-methylcytosine | 0,018 | 0,035 | ----- |
| SBS5 | Unknown | 0,059 | 0,310 | 0,139 |
| SBS6 | Defective DNA mismatch repair | 0,083 | ----- | ----- |
| SBS11 | Temozolomide treatment | 0,727 | 0,341 | ----- |
| SBS19 | Unknown | 0,112 | 0,074 | ----- |
| SBS20 | Concurrent POLD1 mutation and mismatch repair deficiency | ----- | ----- | 0,441 |
| SBS32 | Azathioprine treatment | ----- | ----- | 0,421 |
| SBS37 | Unknown | ----- | 0,191 | ----- |
| SBS54 | Sequencing artefact | ----- | 0,049 | ----- |
| Total Mutations |  | 6024 | 13563 | 46435 |
| Cosine Similarity |  | 0,997 | 0,991 | 0,940 |
| ID | | | | |
| ID1 | Slippage during DNA replication | 0,211 | 0,122 | 0,261 |
| ID2 | Slippage during DNA replication | 0,789 | 0,393 | 0,739 |
| ID19 | Unknown | ----- | 0,256 | ----- |
| ID23 | Aristolochic acid exposure | ----- | 0,229 | ----- |
| Total Mutations |  | 332 | 1310 | 436 |
| Cosine Similarity |  | 0,995 | 0,961 | 0,936 |
